# T-Cell Factors as Transcriptional Inhibitors: Activities and Regulations in Vertebrate Head Development

**DOI:** 10.3389/fcell.2021.784998

**Published:** 2021-11-24

**Authors:** Johnny Bou-Rouphael, Béatrice C. Durand

**Affiliations:** Sorbonne Université, CNRS UMR7622, IBPS Developmental Biology Laboratory, Campus Pierre et Marie Curie, Paris, France

**Keywords:** Tcf/Lef, transcription, signalization, stem cells, Barhl, forebrain, Wnt

## Abstract

Since its first discovery in the late 90s, Wnt canonical signaling has been demonstrated to affect a large variety of neural developmental processes, including, but not limited to, embryonic axis formation, neural proliferation, fate determination, and maintenance of neural stem cells. For decades, studies have focused on the mechanisms controlling the activity of β-catenin, the sole mediator of Wnt transcriptional response. More recently, the spotlight of research is directed towards the last cascade component, the T-cell factor (TCF)/Lymphoid-Enhancer binding Factor (LEF), and more specifically, the TCF/LEF-mediated switch from transcriptional activation to repression, which in both embryonic blastomeres and mouse embryonic stem cells pushes the balance from pluri/multipotency towards differentiation. It has been long known that Groucho/Transducin-Like Enhancer of split (Gro/TLE) is the main co-repressor partner of TCF/LEF. More recently, other TCF/LEF-interacting partners have been identified, including the pro-neural BarH-Like 2 (BARHL2), which belongs to the evolutionary highly conserved family of homeodomain-containing transcription factors. This review describes the activities and regulatory modes of TCF/LEF as transcriptional repressors, with a specific focus on the functions of *Barhl2* in vertebrate brain development. Specific attention is given to the transcriptional events leading to formation of the Organizer, as well as the roles and regulations of Wnt/β-catenin pathway in growth of the caudal forebrain. We present TCF/LEF activities in both embryonic and neural stem cells and discuss how alterations of this pathway could lead to tumors.

## Introduction

Understanding how the vertebrate nervous system emerges from a homogeneous layer of neuroepithelial cells, the neural plate, has long been a subject of intense fascination. Fate-mapping experiments performed at the end of the 20th century demonstrated that the primordia of the forebrain, midbrain, hindbrain, and spinal cord are all already established along the antero-posterior (AP) axis when the neural plate emerges. These studies revealed that a construction blueprint of the neural organization, and specifically that of the forebrain, is set up during gastrulation (reviewed in Wilson and Houart, 2004; Hoch et al., 2009; Andoniadou and Martinez-Barbera, 2013). In 1924, Hans Spemann and Hilde Mangold discovered that the dorsal lip of a newt blastopore, when grafted into the ventral part of a host embryo, is able to induce a secondary axis containing a complete nervous system (Spemann and Mangold, 1924). This small group of specialized cells, referred to as the “Organizer,” emerges during embryonic development at gastrulation, acts as a local source of secreted signaling factors and drives both neural induction and patterning of the prospective neuroepithelium and thereby of the developing head (reviewed in De Robertis et al., 2000; Niehrs, 2004; Kiecker and Lumsden, 2012; Anderson and Stern, 2016). Since this discovery, an organizing center has been found in other model organisms: Hensen’s node in the chick, the node in the mouse and the shield in zebrafish, and the capacity of the blastopore-associated tissue to induce naïve cells to form a fully developed twin embryo was found conserved in non-bilaterian metazoan species (Kraus et al., 2016).

Initial regionalization of the neural plate relies on the synergistic action of at least five major signaling pathways that convey spatial and temporal information to naïve cells, consequently inducing developmental programs that drive their behavior (reviewed in Stern, 2002; Wessely and De Robertis, 2002; Ozair et al., 2013). Amongst the cell-to-cell signaling pathways coordinating development, one of the most conserved in the animal kingdom is the Wnt/β-catenin, or canonical pathway. During emergence of the central nervous system, Wnt/β-catenin acts in a coordinated manner with Sonic HedgeHog (Shh), Notch, Transforming Growth Factor (TGF-β), and Fibroblast Growth Factor (FGF) pathways and contributes to neural patterning, proliferation, and fate determination. Notably, the Wnt/β-catenin machinery drives the transcriptional events leading to the induction of the Organizer, and thereby formation of the embryonic axes. Its participation is also crucial in Neural Stem Cell (NSC) maintenance and self-renewal. Not surprisingly, dysregulation of Wnt/β-catenin signaling is linked to serious brain developmental defects, including cancer (reviewed in Hoppler and Moon, 2014; Brafman and Willert, 2017; Nusse and Clevers, 2017).

After four decades of intense research following the initial discovery of Wnt signals (Nusse and Varmus, 1982, reviewed in Nusse and Varmus, 2012), 19 ligands have been characterized in mammals, together with two families of receptors comprising 10 Frizzled receptors, and two Low-Density Lipoprotein (LDL) receptor-related proteins (LRP5/6) (reviewed in MacDonald et al., 2009; Niehrs, 2012). Despite this complexity, the large majority of Wnt/β-catenin transcriptional targets are regulated by T-Cell Factor/Lymphoid Enhancer-binding Factor (TCF/LEF) transcription factors (TF). Loss of function analysis performed in invertebrate such as the nematode *Caenorhabditis elegans (C. elegans)* and in flies, where a single TCF/LEF has been characterized, provided evidence that TCF/LEF act through a transcriptional switch, which either activates or represses Wnt/β-catenin target genes’ expression. This feature has been further validated in vertebrate, whose genome contains four TCF/LEF members: TCF1, TCF3, TCF4, and LEF1. In this review, the vertebrate TCF/LEF members will be referred to as TCF7 (previously TCF1), TCF7L1 (TCF3), TCF7L2 (TCF4) and LEF1 following the Human Genome Organization (HUGO) nomenclature (Table 1). As will be discussed below, some of the vertebrate TCF/LEF have a more specialized function compared to their invertebrate counterparts.

**TABLE 1:** TCF/LEF, Gro/TLE and BARHL homologues across species.

	***Drosophila melanogaster***	***Caenorhabditis elegans***	***Xenopus laevis***	***Mus musculus***	***Homo sapiens***
TCF/LEF	Pan	POP1	Tcf7 (Tcf1)	TCF7 (TCF1)	TCF7 (TCF1)
			Tcf7l1 (Tcf3)	TCF7l1 (TCF3) TCF7l2 (TCF4)	TCF7l1 (TCF3) TCF7l2 (TCF4)
			Tcf7l2 (Tcf4)	LEF1 (LEF1)	LEF1 (LEF1)
			Lef1 (Lef1)		
Gro/TLE	Gro	UNC-37	Gro1-4	GRG1-4, GRG5	TLE1-4
BARHL1	BarH2	CEH30	Barhl1	BARHL1 (MBH2)	BARHL1
BARHL2	BarH1		Barhl2	BARHL2 (MBH1)	BARHL2

*Invertebrate have a single T-cell factor/Lymphoid enhancer-binding factor (TCF/LEF): Pangolin (Pan) in flies and POP1 in worm. Vertebrate have four TCF/LEF known as TCF7, TCF7l1, TCF7l2 and LEF1, previously termed TCF1, TCF3, TCF4 and LEF1 respectively*. Drosophila *Groucho (Gro) and* C. elegans *UNC-37 corepressors have four vertebrate orthologs: Gro1-4 in frogs, Groucho-related gene (GRG1-4) in mice, and Transducin-like enhancer of split (TLE1-4) in human. In mice, GRG5 acts as a dominant negative. The homeobox-containing proteins BarH1 and BarH2 have been first identified in* Drosophila. *Homologues have been described in vertebrate, named BarH-like or BARHL. In mice, BARHL have been previously referred to as mammalian BarH (MBH1 and MBH2)*. C. elegans *Bar homeodomain gene (CEH30) represents the homologue of* Drosophila *BarH1 and BarH2 and their vertebrate counterparts.*

In most of the species investigated so far, TCF/LEF activate transcription in Wnt-stimulated cells by interacting with the sole transcriptional activator β-catenin (Schuijers et al., 2014), but TCF/LEF can recruit other partners to the transcriptional activating machinery. A massive effort has been deployed to understand TCF/β-catenin transcriptional modes of activation. Very elegant and detailed accounts of the current models supporting Wnt-dependent transcriptional activation events have been published recently (reviewed in van Amerongen and Nusse, 2009; Wiese et al., 2018; Söderholm and Cantù, 2021).

In the absence of Wnt ligands, TCF/LEF interact with repressor partners to inhibit Wnt target genes’ expression. The best-characterized co-repressor partner is Groucho/Transducin-Like Enhancer of split (Gro/TLE) (Brantjes, 2001). Under certain conditions, Gro/TLE can recruit Histone Deacetylases (Hdac) to the complex, a chromatin remodeling enzyme which removes acetyl groups from the N-terminal lysine residues of the core histones, inducing gene expression silencing through chromatin condensation (Sekiya and Zaret, 2007). Recent evidence reveals a role for the homeodomain (HD)-containing TF BarH-Like Homeobox-2 (BARHL2) in enhancing TCF/Gro repressive activity *in vitro* and *in vivo* and preventing the β-catenin-mediated transactivation of TCF/LEF target genes (Sena et al., 2019). These data highlight a novel mechanism regulating Wnt/β-catenin transcriptional response, probably involving the chromatin modifier Hdac1. Studies from hemichordates to vertebrate, which are evolutionarily more than 500 million years apart, have revealed that, despite the differences between species, they all carry two *Barhl* genes: *Barhl1* and *Barhl2*, each having a remarkably evolutionarily conserved structure, distribution, and function. The spectrum of TCF/Gro transcriptional targets is large. Both TCF/LEF and Gro/TLE proteins interact with other TFs, and are targets for developmental signals, which influence their activities. The extent and importance of TCF repressive roles, and their regulatory modes during embryogenesis are neither fully grasped, nor fully understood.

In this review, we present the activities and regulatory modes of TCF as transcriptional repressors with a focus on the developmental roles of *Barhl2*. Specific attention is given to the transcriptional events leading to the formation of the Organizer, as well as the roles and regulations of the Wnt/β-catenin pathway in the growth of the caudal forebrain. We present core activities of TCF/LEF in Embryonic Stem Cells (ESCs) self-renewal and pluripotency, and maintenance of NSCs, as well as their identified deregulations and the emergence of cancer.

### Transcriptional Regulation of Wnt Target Genes by the TCF/LEF Factors – A Focus on the TCF-Mediated Transcriptional Repression

**FIGURE 1:**
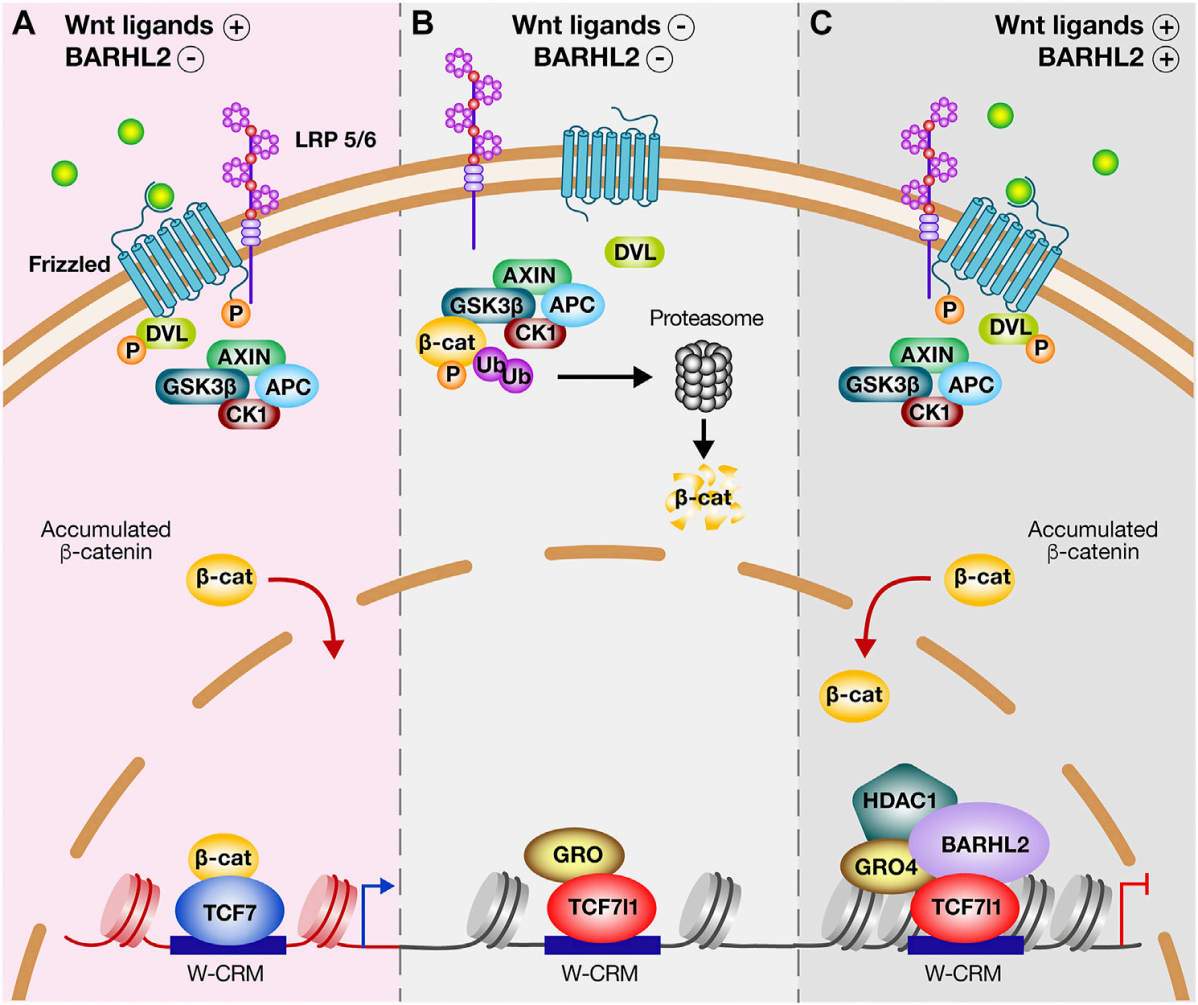
BARHL2 regulatory mode of Wnt canonical pathway. **(A)** Upon Wnt binding to Frizzled (Frz) and Low-Density Lipoprotein Receptor-related Protein (LRP) family of receptors 5 and 6, the multiprotein destruction complex components (Scaffold protein AXIN, Adenomatous Polyposis Coli (APC) tumour suppressor protein, Casein Kinase 1 (CK1), and Glycogen Synthase kinase 3β (Gsk3β)) are recruited to the receptor complex, where they are internalized. Frz binds to Disheveled (DVL) keeping AXIN and Gsk3β inactive. β-catenin (β-cat) escapes degradation, accumulates and translocates into the nucleus, where it binds to activating T-Cell Factors such as TCF7. TCF7 bound on Wnt-Cis regulatory motif (W-CRM) acts as transcriptional activator. **(B)** In the absence of Wnt ligands, the destruction complex is activated. Gsk3β and CK1 phosphorylate β-cat, allowing for its recognition by the E3 ubiquitin ligase (Ub) and targeting it for ubiquitination and proteasomal degradation. In the nucleus, the co-repressive factor Groucho (Gro) binds through its Glutamine (Q)-rich domain to TCF7l1 inducing a transcriptional repression. **(C)** In Wnt-stimulated cells, the presence of BARHL2 inhibits the cell response to β-cat. BARHL2 interacts with the Tryptophan/Aspartic acid (WD)-rich domains of Gro4 *via* its Engrailed Homology 1 (EH1) motifs and interacts with TCF7l1. The domain mediating BARHL2-TCF7l1 interaction is unknown. BARHL2 stabilizes the TCF7l1/Gro4 complex, reinforcing transcriptional repression of Wnt target genes. The complex containing TCF7l1, Gro, and BARHL2 could recruit histone deacetylases (HDAC), which induces inherited epigenetic modifications.

TCF/LEF proteins are the major mediators of Wnt-responsive gene transcription in the nucleus. In the absence of Wnt ligands, β-catenin is phosphorylated by the destruction complex containing Glycogen Synthase Kinase 3β (GSK3β), Casein Kinase 1 (CK1), Adenomatous Polyposis Coli (APC) tumour suppressor protein, and Axin. Phosphorylated β-catenin is targeted towards ubiquitination and further proteasome-mediated degradation. In the nucleus, inhibitory TCF/LEF members are bound on Wnt Cis-Regulary-Motifs (W-CRM), interact with co-repressors such as Gro/TLE proteins, and act as transcriptional repressors. Conversely, in Wnt-stimulated cells, the destruction complex is inhibited leading to the cytoplasmic accumulation of β-catenin and further nuclear translocation. Increase in the nuclear β-catenin levels transiently converts TCF/LEF into transcriptional activators (Figures 1A,B) (reviewed in MacDonald et al., 2009).

Observations from mammalian cells (Schuijers et al., 2014), flies (Franz et al., 2017), and amphibian (Nakamura et al., 2016) among others, reported the requirement of TCF/LEF for the transcriptional regulation of most β-catenin target genes, supporting the classical model of Wnt transcriptional regulation. Mammalian cells lacking all four genes encoding TCF/LEF proteins display perturbations in the association of β-catenin with DNA. In such cells, β-catenin was found to regulate different transcriptional targets (Doumpas et al., 2019), revealing that only when TCF/LEF is absent, β-catenin autonomously regulates a subgroup of genes whose transcription does not initially require TCF/LEF. Genome-wide analysis methods identified Wnt/TCF target genes that are available at http://www.stanford.edu/group/nusselab/cgi-bin/wnt/.

### TCF/LEF Members and Structure

TCF/LEF sequence alignment and phylogenetic trees in species such as the hemichordate *Saccoglossus kowalevskii (S. kowalevskii)*, *Caenorhabditis elegans (C. elegans)*, *Drosophila melanogaster*, *Hydra magnipapillata*, and *Ciona intestinalis* reveal the presence, in the TCF/LEF structure, of the four major binding domains found in vertebrate (Atcha et al., 2007; Žídek et al., 2018), indicating that the TCF/LEF in invertebrate is probably the ancestral precursor of that described in vertebrate. Further complexity has been added through evolution following the emergence of the different TCF/LEF isoforms in mammals which are generated through alternative transcription, translation start sites, and alternative splicing (reviewed in Hoppler and Waterman, 2014).

**FIGURE 2:**
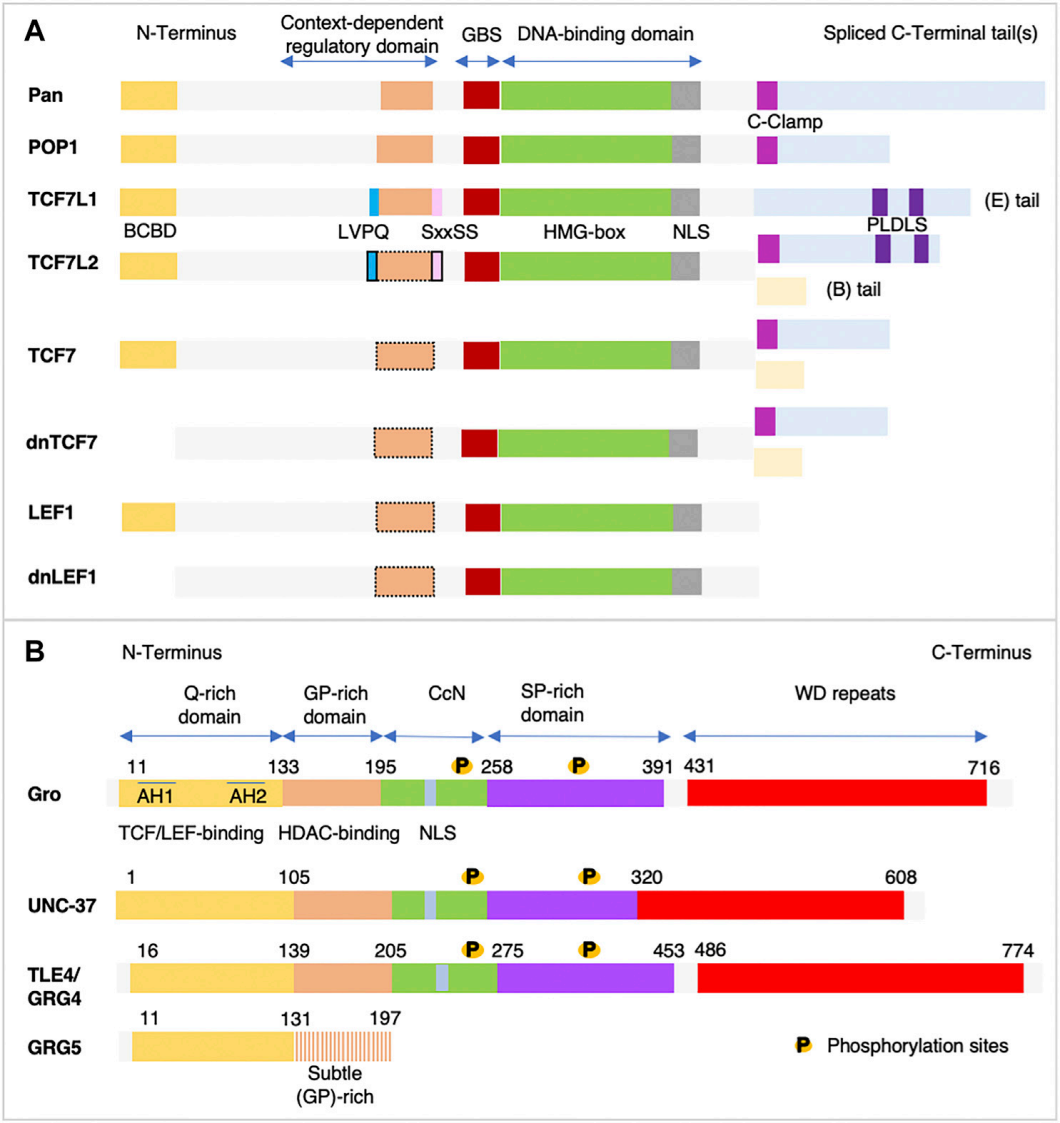
**(A)** Structural organization of the TCF/LEF proteins. Invertebrate (Pangolin (Pan) in *Drosophila*, POP1 in *C. elegans*), and vertebrate T-Cell Factor/Lymphoid Enhancer Factor (TCF/LEF) proteins share several highly conserved domains: the N-terminal β-catenin-binding domain (BCBD) shown in dark yellow, the DNA-binding domain which contains a High-Mobility Group box (HMG-box) shown in green and a Nuclear Localization Signal (NLS) shown in grey. The DNA-binding domain is preceded by a less well-defined binding sequence for the Groucho/Transducin-like enhancer of split (Gro/TLE) (Gro-binding sequence, GBS) shown in dark red. Several protein isoforms are encoded by the genome of vertebrate, except for TCF7l1. The Context-dependent Regulatory Domain (CRD) is less conserved and is encoded by three exons. One of them, indicated in orange, can be alternatively spliced in all vertebrate isoforms except *TCF7L1*. Black dotted lines indicate possible alternative splicing. In TCF7l1 and in TCF7l2, this CRD domain is flanked by two small motifs LVPQ shown in blue at its N-terminal end, and SxxSS shown in pink at its C terminal end, which contribute to TCF7L1/2 repressive activity. Whereas these two motifs are always present in TCF7l1, in TCF7l2 they can be alternatively spliced. Less conservation is found in the C-terminal tail, as it is highly variable in length. The long (E) tails shown in light blue contain additional regulatory domains: the C-clamp DNA-binding motif shown in violet, present in most invertebrate and in the vertebrate TCF7l2 and TCF7, and two C-terminal-binding protein (CtBP) motifs (PLDLS) shown in dark blue, found in TCF7l1 and TCF7l2. Isoforms of TCF7 and LEF1 expressed from alternative promoters and encoding proteins lacking BCBD behave as dominant-negative. Alternative splicing also generates proteins with short (B) tails shown in light yellow. LEF1 isoform lacks the C-terminal tail. This scheme is inspired by (Hoppler and Waterman, 2014). **(B)** Schematic representations of Gro/TLE proteins functional domains. Sequence comparison of the *Drosophila* Groucho (Gro) (NP_001247309.1), human TLE4 (NP_001269677.1), mouse Groucho-related gene 4 (GRG4) (NP_001289876.1), and *C. elegans* UNC-37 (NP_491932.1) reveals the presence of five domains. The two most highly conserved domains are: 1- the amino-terminal glutamine-rich (Q) domain shown in dark yellow that contains two amphipathic α-helices (AH1 and AH2) and is required for Gro/TLE oligomerization, and interactions with other proteins including the TCF/LEF proteins; and 2- the Tryptophane/Aspartic acid (WD)-repeats shown in red that mediates protein-protein interactions, such as those with the Engrailed Homology (EH1)-containing proteins. The central portion of Gro/TLE is less well conserved and contains a Glycine/Proline-rich (GP) domain implicated in the recruitment of the *Drosophila* Rpd3/mammalian Histone Deacetylase (HDAC) shown in orange, a central portion (CcN) domain shown in green containing a Nuclear Localization Signal (NLS) shown in grey, and a Serine/Proline-rich (SP) domain shown in violet. Phosphorylation sites are found in both the CcN and SP domains. Numbers indicate the positions of the boundary amino acids (aa). GRG-5 only contains the Q-rich domain and a GP-rich domain with aa differences compared to the long forms of Gro/TLE, which impede its ability to interact with HDAC. GRG-5 acts as a dominant negative.

Structural and functional analysis of TCF/LEF provided important cues on the domains mediating their transcriptional activities (Figure 2A). On their N-terminal region, all TCF/LEF isoforms have a β-catenin-binding domain (BCBD), which contains 50 amino acids (aa). Three sets of aa are involved in the TCF/LEF-β-catenin interactions: residues 2–15 (known as the β-hairpin module) fit into the groove of the central Armadillo (Arm) repeat domain (the homologue of the vertebrate β-catenin and signal transducer of wingless (Wg) signaling in flies). Residues 16–29 form an extended strand, and residues 40–51 form an α-helix (Graham et al., 2000). TCF/LEF interaction with β-catenin is necessary for their activity (Kratochwil, 2002). Isoforms of TCF7 and LEF1 expressed from alternative promoters, and encoding proteins lacking BCBD, behave as dominant-negative (van de Wetering et al., 1997; Hovanes et al., 2001). Recognition of the specific DNA sequence motif (CCTTTGAT(G/C)) by TCF/LEF is mediated by a highly conserved High Mobility Group (HMG)-box, whose DNA-binding domain structure and general mechanisms of DNA binding and bending, have been extensively studied (van Beest et al., 2000; reviewed in; Malarkey and Churchill, 2012). This HMG-box is followed by a Nuclear Localization Signal (NLS). The BCBD and the DNA-binding domain are separated by a less conserved context-dependent regulatory domain (CRD), partly encoded by an exon (exon VI), which can be alternatively spliced in all TCF/LEF except TCF7L1. Two conserved aa motifs, LVPQ and SxxSS, flank exon VI in *Xenopus* Tcf7l1 and Tcf7l2, but not in Tcf7 or Lef1, and can be alternatively spliced in Tcf7l2. Mutations in these two motifs validate their strict requirement for Tcf7l1 repressive activity. Furthermore, their insertion into the *lef1* sequence abolishes Lef1 activator capacity, as detected through its inability to induce an ectopic secondary axis when injected ventrally in *Xenopus* embryos (Pukrop et al., 2001; Gradl et al., 2002; Liu et al., 2005). Other studies suggest that the Gro/TLE-binding domain encompasses the entire CRD and part of exon VII. Indeed, alternative splicing within the CRD (exon V to exon VII) modifies the interactions of TCF/LEF with Gro/TLE (Young et al., 2019) (reviewed in Hoppler and Waterman, 2014). The C-terminal tail is the most variable region among the TCF/LEF, where much of the aa sequence exhibit a low level of conservation. The C-terminal tail exists either as a long C-terminal extension, referred to as E tail, that contains additional domains, or as a short C-terminal extension, referred to as B tail, lacking the additional transcriptional regulators’ binding domains. Whereas TCF7L1 only carries an E tail, the *LEF1* gene lacks the E-tail-encoding exon (Atcha et al., 2003). The E-tails encode two copies of a specific short motif (PLDLS) that binds the evolutionarily conserved co-repressor phosphoprotein C-terminal-Binding Protein (CtBP). Indeed, CtBP binds to both Tcf7l1, and the Tcf7l2 isoforms carrying an E tail (Brannon et al., 1999; Valenta et al., 2003; Fang et al., 2006). An additional small, highly conserved 30 aa motif (CRARF) is present in invertebrate TCF/LEF and in vertebrate splice variants Tcf7-E and Tcf7l2-E, but not in Tcf7l1. CRARF is required for the β-catenin-mediated transcriptional activation of the *lef1* promoter, and forms a C-Clamp (Cysteine-rich domain) that allows TCF/LEF to bind an additional DNA motif known as the Helper site (5′-RCCGCCR-3′) (Atcha et al., 2007; Ravindranath and Cadigan, 2014).

### A Brief Picture of the Evolution of the TCF/LEF Family

TCF/LEF are metazoan inventions (Adamska et al., 2010). In choanoflagellates, which are unicellular eukaryotes considered the closest known relatives to metazoans, there is no evidence supporting the existence of any TCF/LEF protein and the only found component of the Wnt pathway is GSK3 (King et al., 2008). In invertebrate genome, only one *Tcf/Lef* gene is detected (reviewed in Hoppler and Waterman, 2014). One exception is found in the phylum of Platyhelminthes, in which five *Tcf/Lef* have been found in the genome of the flatworm *Schmidtea mediterranea*. Only two of these Tcf/Lef have a putative BCBD, which suggests a function in mediating Wnt transcription (Brown et al., 2018).

Most of our knowledge about TCF/LEF activity in invertebrate derives from studies performed in *Drosophila* and *C. elegans*. As in vertebrate, their Tcf/Lef is converted from a transcriptional repressor to activator by increasing nuclear levels of β-catenin. *Drosophila* Arm/β-catenin promotes transcriptional activation by binding Pangolin (Pan), the Tcf/Lef in fly (referred to as Tcf) (van de Wetering et al., 1997). Consequently, co-repressors such as Gro are displaced, allowing Arm binding to transcriptional co-activators such as Pygopus (Pygo) (Parker et al., 2002). As in vertebrate, in the absence of Arm, Tcf acts as a transcriptional repressor (Cavallo et al., 1998). Transcriptional repression appears to be directly mediated by the Tcf/Arm interactions with a specific DNA sequence motif (AGAWAW). The exchange of the AGAWAW motif into a standard Tcf-binding site (CCTTTGAT(G/C)) reversed the mode of regulation, resulting in Wnt-mediated activation instead of repression. Whereas both transcriptional activation and repression require binding of Arm to the N-terminal part of Tcf, allosteric regulation has been proposed to explain differences in Tcf/Lef transcriptional capacity. Indeed, Tcf binding to different DNA motifs may allow its interaction with distinct co-regulators, which subsequently controls its transcriptional activity (Blauwkamp et al., 2008).

In *C. elegans*, loss-of-function phenotypes indicate a dual regulatory mode for the Tcf/Lef termed POsterior Pharynx defect (POP1). An interesting mechanism has been reported for mesoderm and endoderm fate specification during embryogenesis (Rocheleau et al., 1997; Thorpe et al., 1997). At the four-cells stage, two sister cells, the anterior (MS) and the posterior (E) are fated to respectively generate the mesoderm and the endoderm. Higher levels of *pop1* are detected in the MS blastomere (Lin et al., 1995), where it represses the transcription of Wnt-responsive endoderm-determining gene *end1* through the recruitment of the histone deacetylase HDA-1 and UNCoordinated (UNC)-37 (the homologue of the Gro/TLE) (Calvo et al., 2001). In a POP1 mutant, both blastomeres adopt an endoderm-like fate. However, in the E blastomere receiving Wnt signals, WRM1/β-catenin binds to the N-terminal domain of POP1 protein and decreases its nuclear levels, alleviating POP1 repressive activity, which will then activate the expression of *end1* and induce the specification of the endodermal fate (Rocheleau et al., 1997; Shetty et al., 2005). Another model proposes that the switch of POP1 from a transcriptional repressor to an activator depends on its DNA-binding site. The C-terminal tail of POP1 contains a C-clamp, which enables POP1 to recognize another DNA motif (the Helper site). When Wnt signaling is activated, β-catenin stabilizes the interaction between the C-Clamp of POP1 and the Helper sites found in the *end1* sequence, which enables *end1* transcription (Bhambhani et al., 2014).

### The vertebrate TCF/LEF are somewhat specialized in transcriptional activation or repression

In vertebrate, the founder members of the TCF/LEF family are TCF7 (van de Wetering et al., 1991) and LEF1 (Travis et al., 1991), initially identified as lymphocyte-regulators in mice. The two other members, TCF7l1 and TCF7l2 have been characterized few years later (Castrop et al., 1992).

TCF/LEF are largely expressed during vertebrate embryogenesis in some overlapping but also distinct regions including the central nervous system, suggesting a functional redundancy of the TCF/LEF members. For instance, in mice, *Tcf7l2* and *Lef1* transcripts are detected in the mesencephalon and the diencephalon (Korinek et al., 1998). In zebrafish, *tcf1* and *lef1* expression overlaps in the tail bud, fin buds and paraxial mesoderm (Veien et al., 2005). Observations made in lung epithelial progenitors also supports redundant and additive functions between the different TCF/LEF members (Gerner-Mauro et al., 2020). However, genetic mutants lacking a single *Tcf/Lef* gene, as well as double knockout (KO) mutants, exhibit severe developmental alterations (van Genderen et al., 1994; Galceran et al., 2000), indicating expanded and diversified roles for each TCF/LEF. Based on these findings among others, a specific activity as Wnt transcriptional activator and/or repressor has been attributed to each TCF/LEF.

*Lef1* and *Tcf7l2* KO mice show reduced Wnt transcriptional activity and are considered to mostly act as activators of the pathway (Korinek et al., 1998; Kratochwil, 2002). Similarly, analysis in zebrafish reveals activating functions for Tcf7, Lef1, and Tcf7l2. Loss of Lef1, expressed in several embryonic tissues, specifically the neural crest, decreases β-catenin activity (Dorsky et al., 1999, 2003). Additional observations from Tcf7l2 mutants show that it maintains proliferation of the intestinal epithelium through activating Wnt target genes’ transcription (Muncan et al., 2007).

In contrast, numerous studies strongly argue that Tcf7l1 mediates Wnt repressive activity. Mice depleted of *Tcf7l1* gene phenocopy those with ectopic activation of Wnt signaling, suffering severe forebrain abnormalities in addition to perturbations in the midbrain and hindbrain (Merrill et al., 2004). Similarly, the zebrafish genome contains two *tcf7l1* genes, *headless hdl*/*tcf7l1a* (Kim et al., 2000) and *tcf7l1b* (Dorsky et al., 2003), giving a total of five *tcf*/*lef* genes. The two Tcf7l1 appear to normally act as transcriptional repressors. *hdl*/*tcf7l1a* mutants exhibit truncated Tcf7l1 protein, which cannot undergo nuclear translocation. Such mutants show severe head defects including a lack of eyes, forebrain, and a part of the midbrain, a hallmark of Wnt overactivation. This phenotype could be rescued by overexpressing *tcf7l1b*, which in this context also act as a negative regulator of the Wnt pathway (Dorsky et al., 2003). Compared to zebrafish, the medaka genome contains a single *tcf7l1* gene. Medaka lacking *tcf7l1* have the same phenotype as the double-mutant zebrafish *hdl*/*tcf7l1b* (Doenz et al., 2018).

Some of the most informative studies regarding transcriptional activities of the four TCF/LEF members came from investigating the development of Spemann organizer (SO) in the amphibian *Xenopus* (also *see* next section). The early *Xenopus* embryo expresses three maternally inherited *tcf*/*lef* mRNAs: *tcf7*, *tcf7l1* and *tcf7l2* (Molenaar et al., 1998; Houston et al., 2002; Roël et al., 2002). Tcf/Lef activities are not redundant during mesoderm induction in amphibian. At late blastula/early gastrula stages, maternally encoded *tcf7l1* represses the dorsal organizer genes’ expression (Houston et al., 2002), whereas both *tcf7* and *tcf7l2* act as transcriptional activators of SO genes (Standley et al., 2006). In this developmental context, whereas an activating form of *tcf7l1* can rescue the Tcf7-morphant phenotype, only a constitutive repressor form of *tcf7l1* rescues the Tcf7l1-morphant phenotype (Liu et al., 2005). Taken together, these observations indicate that during early *Xenopus* mesoderm induction, Tcf7l1 is mostly required for transcriptional repression, whereas Tcf7 and Tcf7l2 mostly mediate transcriptional activation. Interestingly, *lef1* transcripts are first detected after the mid-blastula transition (MBT) (Molenaar et al., 1998), and during mesoderm induction, Lef1 transcriptional activity appears to be redundant with that of Tcf7 (Liu et al., 2005).

### The Interaction Between TCF/LEF and Gro/TLE: A Partnership at the Core of TCF Inhibitory Activity

All the TCF/LEF members need to bind with nuclear co-factors to regulate target genes’ transcription. A key insight into the mechanism of Wnt transcriptional inhibition mediated by the TCF was the finding that they can directly bind to members of the Gro/TLE family of transcriptional co-repressors.

### Structure and Interactions of Gro/TLE Co-repressors

Gro/TLE are evolutionary conserved nuclear proteins. The invertebrate genome encodes a single member: Gro, initially identified in *Drosophila*, and UNC-37 in *C. elegans*, both of which antagonize signaling by Wnt (Cavallo et al., 1998; Calvo et al., 2001). Four members have been identified in human, known as TLE1-4, and in mice, named the Groucho-Related Genes (GRG1-4) (reviewed in Jennings and Ish-Horowicz, 2008; Turki-Judeh and Courey, 2012). In mice, a fifth family member (GRG-5) has also been identified as a gene encoding a shorter variant. GRG-5 is thought to act as a naturally occurring dominant negative (Table 1) (Brantjes, 2001; Wang et al., 2004).

A conserved structural organization comprising five domains characterizes Gro/TLE proteins (Figure 2B). Lacking a DNA-binding domain, Gro/TLE rely on their interaction with transcription factors for their specific recognition of promoter and/or enhancer DNA sequences. The highly conserved N-terminal glutamine-rich (Q) domain contains two motifs termed the amphipathic α-helices (AH1 and AH2), which mediate both Gro/TLE homo-oligomerization and their interactions with various transcription factors, including TCF/LEF (reviewed in Jennings and Ish-Horowicz, 2008). The central portion of Gro/TLE contains three less well-conserved domains. Gro/TLE was found to bind to the *Drosophila* Hdac known as Rpd3 (Chen et al., 1999), and with the mammalian HDAC1 an interaction mediated by the glycine (G) and proline (P)-rich domain (GP) (Chen et al., 1999; Arce et al., 2009). Second, the central (CcN) domain which includes a NLS, and third, a Serine (S) Proline (P)-rich domain (SP) generally involved in repression. The CcN and SP domains contain phosphorylation sites, which can modulate Gro/TLE-mediated repression (reviewed in Jennings and Ish-Horowicz, 2008). Of note, GRG5 contains the TCF/LEF binding domain and a GP domain that carries mutations impeding its ability to interact with HDAC (Brantjes, 2001). At their C-terminal end, Gro/TLE have a four tryptophan-aspartic acid repeat domain (WD), which is highly conserved across evolution. The WD motif is involved in nucleosome binding and condensation (Sekiya and Zaret, 2007), and mediates Gro/TLE interactions with repressor proteins. The WD motif of Gro/TLE interacts with two distinct peptidic motifs, the Engrailed Homology-1 (EH1) motif, and the WRPW (Trp-Arg-Pro-Trp) motif.

The EH1 motif is a Phenylalanine/Isoleucine/Leucine (FIL)-rich domain (FxIxxIL), required for transcriptional repression *in vitro* and *in vivo* (Smith and Jaynes, 1996; Muhr et al., 2001; Jennings et al., 2006). The EH1 motif is found in a large number of HD-containing TFs involved in neuronal specification such as Gastrulation Brain homeobox 2 (GBX2), Orthodenticle homeobox 2 (OTX2) (Heimbucher et al., 2007), Forkhead box (FOX) family of TFs (Yaklichkin et al., 2007), Engrailed (EN) (Jimenez et al., 1997) and BARHL that are notably the only Gro/TLE partners containing two EH1 domains (Offner et al., 2005). The TF Dorsal is involved in DV axis patterning in *Drosophila*. Dorsal was found to physically interact with Gro. Interestingly, in embryos lacking Gro, Dorsal functions as a transcriptional activator rather than as a repressor (Dubnicoff et al., 1997). It has been demonstrated that Gro interacts with a motif with partial homology to the EH1, located in the C-terminal part of Dorsal (Flores-Saaib et al., 2001). This interaction is weak and is stabilized by the presence of additional Gro-binding repressors (Valentine et al., 1998).

The second Gro/TLE-interacting motif is the WRPW present in basic-helix-loop-helix (bHLH) proteins including the Hairy/Enhancer of Split (E(spl))/HES proteins, transcriptional repressors that function as downstream targets of activated Notch receptors (Grbavec et al., 1998) (reviewed in Cinnamon and Paroush, 2008; Turki-Judeh and Courey, 2012). In the absence of Notch signaling, Gro/TLE is recruited *via* Hairless to a complex containing Suppressor of Hairless (Su(H)) and CtBP, which represses Notch target genes, including E(spl). Upon activation of Notch signaling, the Notch intracellular domain (NICD) enters the nucleus, displaces the Gro-containing complex, recruits Mastermind (Mam) on Su(H) an interaction which further results in the transcriptional activation of E(spl). E(spl) encoded factors interact with Gro/TLE to repress proneural genes (reviewed in Cinnamon and Paroush, 2008; Turki-Judeh and Courey, 2012). In *Drosophila*, and mammals association of Gro/TLE to bHLH proteins is required in cell fate decisions during tissue development including neurogenesis, segmentation, sex determination and myogenesis (Paroush, 1994; Jimenez et al., 1997). The WRPW motif has been demonstrated to be a functional transcriptional repression domain. It is sufficient to confer active repression to Hairy-related proteins or a heterologous DNA-binding protein through its ability to recruit Gro/TLE to target gene promoters (Fisher et al., 1996). Similar to Dorsal, in *Drosophila*, the Runx family member Lozenge that contains a WRPW motif exhibit low affinity for Gro/TLE and requires the Cut HD protein to form a stable repressive complex (Canon, 2003).

### Gro/TLE Acts as Co-repressor in the Presence and Absence of β-catenin

The Gro/TLE-binding site in the central portion of TCF/LEF extends and overlaps the β-catenin binding site (Daniels and Weis, 2005). Therefore, association of Gro/TLE with TCF/LEF counteracts the TCF/β-catenin transactivation activity (Cavallo et al., 1998; Roose et al., 1998; Brantjes, 2001). Together with other observations, these data lead to the generally accepted model where β-catenin activates Wnt-responsive genes by simply displacing Gro/TLE. Whereas recent studies provide arguments for a more complex regulation of Wnt-driven transcriptional switch (reviewed in Ramakrishnan et al., 2018), a large spectrum of genes are regulated by both β-catenin and Gro/TLE through their respective interactions with TCF/LEF. Chromatin immunoprecipitation sequencing (ChIP-seq) data from *Xenopus* embryos provide over 80% correlation between β-catenin and Gro/TLE-binding sites (Nakamura et al., 2016). In mouse hair follicle stem cells, more than half the genes occupied by TCF/LEF are also occupied by Gro/TLE (Lien et al., 2014).

The way Gro/TLE mediate transcriptional repression is still a matter of debate. Recent observations indicate that Gro/TLE could act either short distance *via* modulating RNA-polymerase II (RNA-Pol II) activity, and/or long distance *via* chromatin remodeling. ChIP-seq analysis combined to RNA-seq data performed in *Drosophila* identified the Gro/TLE direct targets. Such analysis suggested that Gro/TLE doesn’t affect the recruitment of RNA-Pol II to the transcription start sites but further increases RNA-Pol II pausing time (Kaul et al., 2014). Other studies indicate that in some context, Tcf/Gro complex promotes compaction of the chromatin when the canonical Wnt pathway is switched off. As previously mentioned, Gro/TLE interact with Hdac. In the presence of an Hdac-inhibitor, Wnt target genes are de-repressed (Billin et al., 2000). It is therefore possible that Gro/TLE interaction with Hdac drives long distance, transmittable changes in the chromatin state. Other studies argue that Hdac recruitment does not account for full co-repressor activity, suggesting that another Gro/TLE-dependent silencing could occur *via* tetramerization of Gro on a Tcf7l1/Gro complex, thereby promoting structural transitions of chromatin leading to transcriptional repression (Sekiya and Zaret, 2007; Chodaparambil et al., 2014).

### The Gro/TLE and TCF/LEF interaction(s) in Early Axis Specification

In *Xenopus* embryos, injection of *gro* represses transcription of Wnt target genes (Roose et al., 1998), and mutations in Gro/TLE-binding sites of *tcf7l1* reduces Tcf7l1 repressive activity (Liu et al., 2005; Tsuji and Hashimoto, 2005). Analysis performed on the *Xenopus siamois (sia)* promoter demonstrated that Tcf/Lef-binding sites mediate both basal repression and β-catenin-dependent activation at the W-CRM (Brannon et al., 1997; Fan et al., 1998). More recently, large-scale analysis demonstrates that in the dorsal blastomeres, Gro/TLE binds to the same W-CRM as β-catenin (Yasuoka et al., 2014; Nakamura and Hoppler, 2017; Afouda et al., 2020). In this context a few lines of evidence indicate that β-catenin activates Wnt-responsive genes by displacing the whole Tcf7l1/Gro repressor complex and replacing it with an activator complex, containing β-catenin in association with Tcf7 (Chambers et al., 2017) (reviewed in Cinnamon et al., 2008; Sokol, 2011; Ramakrishnan et al., 2018)

In conclusion, the mechanisms by which Gro/TLE mediate transcriptional repression in the presence and/or absence of TCF/LEF are still not fully understood. To add complexity, both TCF/LEF and Gro/TLE proteins are targets for developmental signals, which influence the affinity of Gro/TLE to TCF/LEF and/or W-CRM. Thereby, the developmental and cellular contexts in which Gro/TLE repression causes epigenetic regulations *via* the binding of HDAC by Gro/TLE as well as the exact role(s) of such transcriptomic regulations during development are still poorly understood.

## Tcf/Lef and Barhl2 in the Developmental Dynamics of Spemann Organizer (SO)

### Both Tcf/Lef Repressor and Activator Functions Are Required for Normal SO Development

One of the earliest, well-documented, and evolutionarily conserved functions of Wnt/β-catenin signaling is the induction of the blastopore lip organizer. The discovery made by Spemann and Mangold in 1924 has revolutionized our understanding of embryonic axis formation. In their classic transplantation experiment in newt, the authors showed that a mesodermal region - the dorsal lip of the blastopore - of a gastrula embryo induces a secondary axis including a complete nervous system when grafted ventrally (reviewed in De Robertis et al., 2000). This primary embryonic organizing center known as SO determines the dorso-ventral (DV) body axis. Requirement of canonical Wnt signaling for axis formation has been demonstrated following overexpression of Wnt signaling components. For instance, in *Xenopus, wnt1* (McMahon and Moon, 1989), *wnt8* (Sokol et al., 1991), and *β-catenin* (McCrea et al., 1993) can induce a complete dorsal axis when overexpressed ventrally. A similar phenotype has been observed when two Wnt inhibitors are depleted: Tcf7l1 (Merrill et al., 2004) and Axin2 (Zeng et al., 1997). More recently, it was shown in non-bilaterian metazoan species that the same molecular mechanism was used for inducing secondary axes as in chordates: the Wnt/β-catenin signaling, indeed demonstrating that the emergence of the Wnt/β-catenin driven blastopore-associated axial organizer predates the cnidarian-bilaterian split, which occurred over 600 million years ago (Kraus et al., 2016).

**FIGURE 3:**
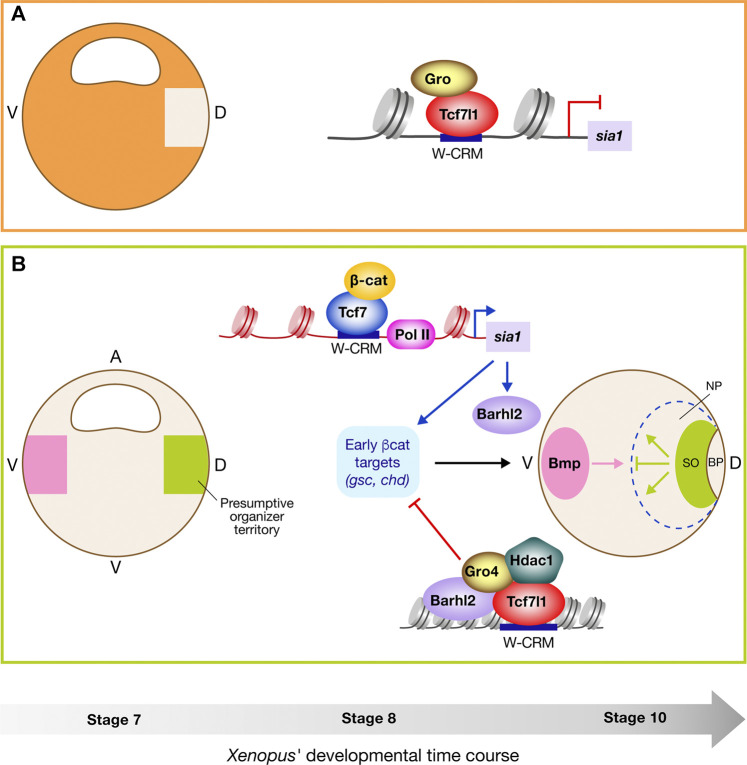
Barhl2 switches off early β-catenin response during establishment of Spemann organizer in Xenopus. **(A)** Maternally encoded Tcf7l1 represses Wnt target genes’ transcription (e.g., *siamois-1 (sia1*)) throughout the entire embryo except dorsally in the presumptive organizer territory starting at stage 7. **(B)** Dorsally (green area), nuclear β-catenin (β-cat) level locally increases allowing its interaction with T-Cell Factor (probably mostly Tcf7 and Tcf7l2), and the initiation of *sia1* transcription. Between stage 8 and 9, Sia1, together with β-cat, induce expression of the dorsal early β-cat target signature including *goosecoid* (*gsc*) and *chordin* (*chd*) leading to the formation of Spemann organizer (SO). Evidence argues that around the same time, *sia1* induces *barhl2* transcription. Barhl2 being a part of a repressive complex together with Groucho-4 (Gro4), Tcf7l1, and Histone deacetylase-1 (Hdac1), switches off the early β-cat dorsal signature *via* an inherited epigenetic regulatory mode thereby limiting SO establishment in time and/or space. SO gives rise to the prechordal plate and the notochord, two tissues that send planar and vertical signals to the overlying prospective neuroepithelium. At stage 10, signals secreted by the SO, including Bone Morphogenetic Protein (Bmp) inhibitors and Wnt signals, enable initiation of the dorsal developmental program: The first blastopore lip cells invaginating into the embryo will give rise to the prechordal plate, followed by the cells that will generate the notochord. Together, the prechordal plate, and the notochord, will send planar and vertical signals that both induce and pattern the overlying neuroepithelium and thereby constitute a secondary organizer (the axial organizer). The prechordal plate plays a major role in inducing and patterning of the anterior neural plate, generating the forebrain and midbrain. The notochord participates in formation of the Sonic hedgehog (Shh)-secreting floor plate and induces and patterns the posterior neural plate (reviewed in Stern, 2002; Wessely and De Robertis, 2002; Niehrs, 2004; Wilson and Houart, 2004; Hoch et al., 2009; Ozair et al., 2013; Brafman and Willert, 2017). V, ventral; D, dorsal; BP, blastopore; NP, neuroepithelium; A, Animal pole; V, Vegetal pole.

Investigations from the past decades lead to the current model of SO development. Before initiation of zygotic transcription, Tcf7l1 represses gene transcription throughout the embryo (Figure 3) (Molenaar et al., 1996; Houston et al., 2002). Accumulation and stabilization of β-catenin by maternal determinants in the nucleus of the dorsal cells, inhibit Tcf7l1 repressors’ activity (Schneider et al., 1996; Larabell et al., 1997), and activate the transcription of s*ia* and *siamois homologue 2 (twin)* (Lemaire et al., 1995; Carnac et al., 1996), which in turn activate the transcription of 123 genes, including *goosecoid (gsc)* and *chordin (chd)*, leading to the formation of the SO territory. All genes de-repressed by β-catenin in this region have been identified (Ding et al., 2017b). *sia* and *twin* are directly regulated by binding Tcf/Lef to their promoters, and poised for transcriptional activation by β-catenin before the Mid blastula transition (MBT) (Brannon et al., 1997; Laurent et al., 1997; Fan et al., 1998; Blythe et al., 2010). In the absence of β-catenin, Tcf7l1, together with Gro/Tle, inhibit *sia* and *twin* transcription (Roose et al., 1998). More recently, a thorough transcriptomic analysis, combined with genome-wide β-catenin association using ChIP-seq, identified stage-specific direct Wnt target genes. The direct comparison of genome-wide occupancy of β-catenin with a stage-matched Wnt-regulated transcriptome reveals that only a subset of β-catenin-bound genomic loci are transcriptionally regulated by Wnt signaling. The differences in classes of direct Wnt target genes appear to be context specific, and dependent on the presence of co-factors such as FoxH1, Nodal/TGFβ signaling (Afouda et al., 2020), Bone Morphogenetic Protein (BMP), and FGF signaling (Nakamura et al., 2016). These studies reveal that the cellular transcriptional responses to Wnt signal are highly dependent on the context, and thereby on the tissue, the developmental steps, the presence of co-factors and/or activation of co-signaling pathways (reviewed in Nakamura and Hoppler, 2017).

### The Evolutionary Conserved BARHL Proteins Interact Independently With Both Gro/TLE and TCF/LEF

**FIGURE 4:**
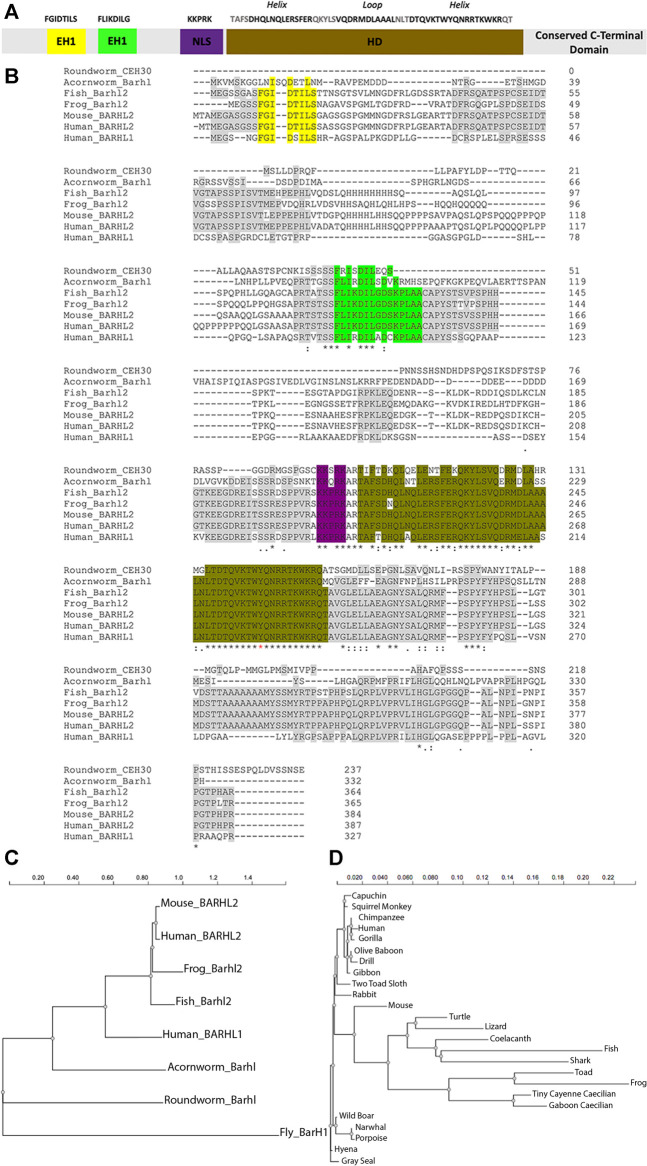
BARHL proteins are highly conserved through evolution. **(A)** Scheme of BARHL1 and BARHL2 proteins. Both proteins share high similarities in their aa sequences. The most conserved regions are the two Engrailed Homology (EH1) domains shown in yellow and green, the homeodomain (HD) shown in brown, the Nuclear Localisation Signal (NLS) shown in violet, and a functionally uncharacterized C-terminal region. **(B)** Multiple sequence alignment of BARHL proteins. Shown is a representative selection of some BARHL2 protein sequences in vertebrate including frog *Xenopus laevis* (NP_001082021.1), fish *Danio rerio* (NP_991303.1), mouse *Mus musculus* (NP_001005477.1) and human *Homo sapiens* (NP_064447.1) among several other vertebrate sharing the same amino acid (aa) sequences, together with the human BARHL1 protein sequence (NP_064448.1), in addition to the invertebrate roundworm *Caenorhabditis elegans* Bar homeodomain CEH-30 (NP_508524.2) and hemichordate acornworm *Saccoglossus Kowalevskii* Barhl (NP_001158386.1). Mouse MBH1 (mammalian BarH1) is referred to as BARHL2. Alignments are generated by ClustalW. Identical aa within the conserved regions are highlighted. EH1 domains (yellow and green) are highly conserved in vertebrate. However, only the second EH1 domain (green) is found in roundworm and acornworm. The most conserved region between vertebrate and invertebrate is the HD (brown), preceded by a NLS (purple). Other conserved aa, specifically those located on the C terminus, haven’t been functionally characterized and are depicted in grey. The HD of BARHL proteins contains a tyrosine (Y) at site 49 (red asterisk), as opposed to phenylalanine (F49) in other homeoproteins. Below the protein sequences is a key denoting conserved sequence (*), conservative mutations (:), and semi-conservative mutations (.). Phylogenetic trees showing evolutionary distance between **(C)** the BARHL protein sequences in invertebrate and vertebrate, and **(D)** a larger selection of vertebrate BARHL2 protein sequences. The Trees were constructed by NGPhylogeny.fr (Lemoine et al., 2019) using FastME2.0 program which provides distance algorithms to infer phylogenies based on the balanced minimum evolution approach. The trees are drawn to scale, which represents the number of differences between sequences through evolution. *Drosophila melanogaster* (NP_001259642.1) shows several divergent regions: fly BarH1 (mammalian BARHL2) protein carries the first EH1 domain and HD but has several additional aa found on the N-terminal and C-terminal parts. BARHL2 protein sequence is similar in all higher vertebrate (less than 2% difference).

The Bar-class HD, BarH1 and BarH2, are HD-containing transcription factors initially discovered in *Drosophila* (Kojima et al., 1991; Higashijima et al., 1992). *Barhl* genes have subsequently been identified in fish (zebrafish, medaka), amphibian (*Xenopus*), birds (chicken), mammals (mouse, human), nematode (*C. elegans*) and *S. kowalevskii* among others (Lowe et al., 2003; Pani et al., 2012; Yao et al., 2016). Phylogenetic analysis shows that BARHL1 and BARHL2 proteins are extremely well conserved in the chordate phylum and are predominantly expressed in the central nervous system (CNS), where their expression patterns are distinct but partially overlapping (Figure 4) (Bulfone, 2000; Patterson et al., 2000; Offner et al., 2005; Colombo et al., 2006) (reviewed in Schuhmacher et al., 2011). BARHL1 and BARHL2 are involved in diverse processes such as the acquisition of a neural identity in the retina, specification of commissural neurons in the spinal cord and cell migration in the cerebellum and the hindbrain (Chellappa et al., 2008; Ding et al., 2009; Jusuf et al., 2012) (reviewed in Reig et al., 2007).

BARHL proteins are characterized by a conserved HD sequence of about 60 amino acids, which forms a three-dimensional helix-loop-helix structure required for their fixation to DNA (Figures 4A,B) (Gehring et al., 1994). Unlike other homeoproteins, BARHLs contain a tyrosine (Y) at site 49 as opposed to phenylalanine (F) at this site of the HD. Whilst the biological significance behind this substitution is unknown; it is thought that there could be a difference in the specificity of the DNA recognition motif. BARHL sequence also contains an NLS, and at their amino-terminal region, two EH1 domains. Sequence comparison reveals a conserved domain with an unknown function at the C-terminal part of BARHL proteins.

Biochemical experiments performed in both mammalian cells and *Xenopus* embryo validate the physical interaction between BARHL2 and Gro/TLE. Surprisingly, BARHL2 was found to interact with TCF/LEF, more specifically TCF7l1, and dramatically enhance the ability of TCF7l1 to co-immunoprecipitate Gro4/TLE4, at least in mammalian cells. This interaction is independent of TCF7L1 binding to Gro/TLE. Functional observations confirm that Barhl2 enhances the capacity of to repress transcription, and abolishes the β-catenin-driven activation of TCF/LEF target genes (Figure 1C) (Sena et al., 2019).

### Barhl2 Normally Limits SO Formation Through Enhancing the Ability of Tcf to Repress Transcription

In *Xenopus, barhl2* is not expressed maternally. Whereas W-CRM have been identified in the *barhl2* loci (Nakamura et al., 2016), *barhl2* is neither part of the early dorsal β-catenin signature, nor induced by overexpression of RNA coding for *wnt8b*. It is however expressed following the initiation of early β-catenin induction, and its expression increases following *sia1* mRNA overexpression (Owens et al., 2016; Session et al., 2016; Ding et al., 2017b; Sena et al., 2019), suggesting that at these developmental stages, *barhl2* transcription is under the control of both *sia1* and β-catenin (Figure 3).

In *Xenopus*, overexpression of *barhl2* generates massive developmental defects including loss of the SO territory and all anterior structures, including the cement gland and the head. In contrast, Barhl2 depletion expands both the organizer territory and its signaling activity, as detected through a massive increase in neuroepithelium size, and patterning alterations (Offner et al., 2005; Sena et al., 2019). Experimental evidence demonstrates that these developmental defects are direct consequences of Barhl2 normally enhancing Tcf7l1-mediated transcriptional repression. These observations lead to a model in which stabilization of β-catenin first de-represses Tcf7l1, and then initiates the dorsal developmental program through activating Tcf7 and/or Tcf7l1. The presence of Barhl2 locks Tcf7l1 and/or Tcf7 in an inhibitory state, and consequently limits induction of the dorsal development program. In this way Barhl2 participates in progression of the blastula development, and normally limits SO formation in time and/or in space.

Analysis of Barhl2 proteins that are mutated either in their ability to interact with DNA, or to bind Gro/TLE, indicate that its normal role requires both. As previously stated, Gro/TLE can silence target genes by tetramerizing on a Tcf7l1-Gro complex (Chen et al., 1999; Chodaparambil et al., 2014). It is therefore possible that Barhl2 enhances the binding of the complex to histones, associated with the long-term silencing of Tcf/Lef target genes through increasing Gro/TLE stoichiometry in a protein complex containing Tcf7l1. Moreover, the presence of Hdac1 is detected in a protein complex containing Barhl2, Tcf7l1 and Gro4. Hdac1 depletion promotes SO development. In parallel, Barhl2 depletion promotes key organizer genes’ acetylation. Thereby, Hdac1 activity could contribute to the Barhl2-mediated repression of Wnt target genes. ChIP-qPCR observations on the promoter of *gsc* indicate that both Barhl2 and Tcf7l1 can interact with the same Tcf-W-CRM in the absence of an adjacent Barhl2-W-CRM.

Overall, these observations are consistent with Barhl2 acting over long distance *via* its specific binding on DNA, perhaps on super-enhancers as previously suggested (Lin et al., 2016), and inducing long-term silencing of SO target genes maybe *via* Hdac1 activity and/or direct interaction with chromatin. In this way Barhl2 irreversibly locks cells in a SO identity.

## In the Diencephalic Primordium, Barhl2 Limits Wnt/Tcf Activity

### Patterning and Growth of the Diencephalic Territory Requires High Levels of Wnt Signals and the Presence of the Morphogen Sonic HedgeHog (Shh)

The forebrain (telencephalon and diencephalon) is derived from the most anterior part of the neuroepithelium: the prosencephalic neural plate. Fate mapping analysis revealed that the telencephalon emerges from the most anterior part of the neural plate, whereas the diencephalon is formed within the caudal forebrain.Whereas inhibition of Wnt pathway is strictly necessary for telencephalic development (Glinka et al., 1998) (reviewed in Wilson and Houart, 2004), growth and patterning of the diencephalic territories (thalamus and epithalamus) require high levels of Wnt. While the *Wnt1* or *Wnt3A* KO mice lose both the midbrain and hippocampal areas, double *Wnt3A/Wnt1* mutant embryos exhibit an additional reduction in the diencephalon, caudal hindbrain, and rostral spinal cord (Thomas and Capecchi, 1990; Lee et al., 2000). Conversely, ectopic expression of *Wnt1* or *Wnt3A* induces the enlargement of the neural tube along the DV axis, without altering the cellular identities of diencephalic neurons (Megason and McMahon, 2002; Panhuysen et al., 2004). Zebrafish *masterblind (mbl)*-mutant embryos carrying a mutation in the GSK3-binding domain of Axin1, which constitutively activates Wnt signaling, show a net reduction in the telencephalic and retinal territories in favor of the diencephalic territory (Heisenberg, 2001). Indeed, the diencephalic primordium, more specifically the diencephalic alar and roof plates, express Wnt ligands such as *Wnt3*, *Wnt3A*, *Wnt8B*, *Wnt4* and *Wnt2B* (Colombo et al., 2006; Juraver-Geslin et al., 2011, 2014; Schuhmacher et al., 2011). Wnt target genes’ expression as well as the Wnt signaling machinery are enriched in the thalamus of all vertebrate analyzed so far (Jones and Rubenstein, 2004; Shimogori et al., 2004; Quinlan et al., 2009; Mattes et al., 2012).

Besides its role in fate determination, Wnt promotes cell-cycle progression, and cell growth. Its ability to modulate the activity of GSK3β promotes a general increase in protein stability, specifically that of β-catenin (Taelman et al., 2010), and through activation of Target of Rapamycin (TOR) pathway, it stimulates growth and protein synthesis. β-catenin nuclear accumulation induces TCF/LEF-mediated expression of the proto-oncogene *c-Myc* (He et al., 1998), which encodes a bHLH leucine zipper (bHLHZip) TF that has two distinct roles in the G1 progression. On one hand, it increases the expression of *CyclinD1* and *CyclinD2* that promotes progression from the G1 to the S phase; on the other, it represses the cell cycle inhibitors *p27Kip1* and *p21Cip1*, thereby promoting cell cycle progression, and enhancing cell proliferation (reviewed in Juraver-Geslin and Durand, 2015).

The Sonic hedgehog (Shh)-secreting Mid-Diencephalic Organizer (MDO), also known as the *Zona Limitans Intrathalamica* (*zli*), develops within the diencephalic primordium (Larsen et al., 2001). Within the thalamic complex, Shh secreted by *zli* cells participates in the survival, growth, and patterning of neuronal progenitor subpopulations (Hashimoto-Torii et al., 2003; Scholpp et al., 2006, 2007; Vieira and Martinez, 2006). Mice lacking *Shh* show severe defects in most of the diencephalic territory (Chiang et al., 1996; Ishibashi and McMahon, 2002). Investigation of the chick neural tube growth revealed an epistatic relationship between Shh and Wnt in progression of the G1 cell cycle phase: Shh permits transcriptional activation of *Tcf7l1* and *Tcf7l2*, which then induces β-catenin dependent expression of *Cyclin-D1* (Alvarez-Medina et al., 2008). Phenotypic observations of *Shh* mutated mice suggest a conservation of these interactions in the diencephalon. Such mice develop a reduced diencephalon with decreased *Tcf7l2* expression (Ishibashi and McMahon, 2002).

### In the Diencephalon, Barhl2 Acts as a Brake on Progenitors’ Proliferation by Limiting Wnt Activity

Shh and Wnt synergistically promote proliferation in the alar diencephalon, whereas cell-cycle analysis in chicken and mice reported slow proliferation kinetics in the diencephalon compared to its neighboring territories (reviewed in Martínez and Puelles, 2000). Moreover, diencephalic changing patterns observed upon manipulation of Wnt activity appear to be primarily due to altered fate specification rather than changes in proliferation (reviewed in Wilson and Houart, 2004). *barhl2* transcripts are detected in the diencephalic histogenic field at late gastrula/early neurula stages in *Xenopus* (Offner et al., 2005; Juraver-Geslin et al., 2011), zebrafish (Staudt and Houart, 2007), and mice (Mo et al., 2004). In the diencephalic anlage, Barhl2 acts upstream of Shh in establishment of the *zli* and its absence generates massive defects specifically in the patterning of the alar diencephalon (Juraver-Geslin et al., 2014; Yao et al., 2016; Ding et al., 2017a) (reviewed in Sena et al., 2016). Besides its role in *zli* formation, Barhl2 normally limits diencephalic progenitors’ proliferation: Barhl2-depleted *Xenopus* embryos exhibit both a dramatic hyperplasia, and a neuroepithelial architectural disorganization in the caudal forebrain (Juraver-Geslin et al., 2011, 2014). In depth analysis of Barhl2-depleted embryos revealed an excessive Wnt transcriptional activation that stimulates neuroepithelial cell proliferation and induces defects in β-catenin intracellular localization together with an upregulation of *axin2* and *cyclinD1*. Measurement of the relative velocity of the cell cycle in Barhl2-depleted embryos reveals a shortening of the cell cycle length (6 versus 8 h). As the length of the S-phase in these cells remains unchanged (1.5 h), and CyclinD1 is part of the G1-S cell cycle checkpoint, Barhl2 probably acts on the length of G1 phase (Juraver-Geslin et al., 2011).

Interestingly, in the developing diencephalon, a non-apoptotic function of the effector caspase, Caspase-3, limits neuroepithelial cell proliferation by inhibiting the activation of Tcf/Lef by the β-catenin (Juraver-Geslin et al., 2011). In this context, Caspase-3 acts either in parallel, or downstream of Barhl2, and its activity does not depend on its apoptosis-effector function. In addition, in the neuroepithelium, Caspase-7 acts as the executioner Caspase leading to cell death (Sena et al., 2020). Indeed, how Barhl2 regulates Caspase-3 non-apoptotic activity in *Xenopus* and limits β-catenin levels and stability in the developing diencephalon is unknown.

In conclusion, in the caudal forebrain, Barhl2 acts as a brake on Wnt transcriptional activation, probably through the stabilization of the inhibitory Tcf/Gro complex. Barhl2 could increase the length of diencephalic progenitors’ G1 phase, thereby modulating neuronal progenitors’ response to extracellular signals, including those of Wnt and Shh.

### Wnt Signals Influence Diencephalic *Barhl2* Expression

What are the extracellular signals influencing *Barhl2* expression and activity in the caudal forebrain? *Wnt3a* is expressed in E9.5 mice (Louvi et al., 2007) at the onset of *Barhl2* expression in the same territories. Pioneer studies performed in *Drosophila* presented the wg pathway as a positive regulator of *barhl2* expression in the notum. *barhl2* expression was lost in clones mutated for Arm (reviewed in Reig et al., 2007). Conversely, the expression of a constitutively active form of *arm* induces an ectopic expression of *barhl2* in the pre-scrutum, associated with a decrease of wg (Sato et al., 1999). In *Xenopus,* RNA-sequencing analysis revealed that both morpholino-mediated depletion of Tcf7l1, and pharmacological activation of Wnt canonical signaling, induce an increase in *barhl2* transcripts (Wills and Baker, 2015; Stevens et al., 2017).

Taken together, these observations suggest a model where Barhl2 could be a direct, or an indirect, target of the canonical Wnt signaling pathway. In return, Barhl2 would establish a negative feedback loop that limits Wnt’ activity.

## Other Regulators of TCF/LEF-Gro/TLE Transcriptional Activity

### Transcription Factors Binding to Tcf7l1 Influence Its Repressor Activity in a Positive And/Or Negative Way

Beside Barhl2, other co-repressors influence Tcf7l1 inhibitory activity. Indeed, cDNA expression screens performed in mammalian cells, combined with functional analysis in *Xenopus*, identified 45 inducers and 96 inhibitors of Tcf/Lef activity (Freeman et al., 2015). Co-repressors’ modes of action are diverse, sometimes divergent between vertebrate and invertebrate, and involve protein–protein interactions, changes in Tcf7l1 affinity for Wnt-target gene promoters, recruitment of co-repressors or co-activators, modulation of protein stability, and nuclear translocation.

CtBP, first described in *Xenopus* and later in rodents and human, binds to the C-terminal part of Tcf7l1-E and Tcf7l2-E isoforms. In fly, CtBP appears to be required for both activation of some Wnt targets and the repression of others, in parallel to, and independently of Tcf/Lef (Fang et al., 2006). However, the vertebrate CtBP acts as a co-factor for Tcf7l1, enhancing its repressor activity (Brannon et al., 1999; Xia et al., 2011). Lack of both Gro/TLE-binding domain and of the C-terminal region of Tcf7l1 leads to target genes’ transcriptional activation (Gradl et al., 2002). Notably, during *Xenopus* SO formation, the C-terminal part of Tcf7l1, which recruits the CtBP, is not required (Liu et al., 2005). In colorectal cancer cells, TCF7l1 recruits both CtBP and HDAC1 to repress expression of the Wnt antagonist DICKKOPF4 (DKK4) (Valenta et al., 2003; Eshelman et al., 2017). Besides CtBP, Tcf7l1 directly interacts with the methyl-CpG-dependent transcriptional repressor Kaiso in *Xenopus*. This interaction results in their mutual disengagement from the respective DNA-binding sites in such a way that Tcf7l1 can be inhibited following Kaiso overexpression both in cell lines, and *Xenopus* embryos (Daniel and Reynolds, 1999). Kaiso cooperates with Tcf7l1 to repress β-catenin target genes such as *sia*, through epigenetic regulation (Park et al., 2005). The interaction of Kaiso with Tcf7l1 depends on Kaiso zinc-finger domains, and on the HMG-box DNA-binding domain of Tcf/Lef factors (Ruzov et al., 2009). The LIM (Lin-11, Islet-1, and Mec-3; the three original members of the family) protein HIC-5 [Hydrogen Peroxide-Induced Clone 5, also termed ARA-55 (Androgen Receptor Activator of 55 kDa)] has been also identified as a binding partner to Tcf7l1 and Tcf7l2. Overexpression of HIC-5 acts as a negative regulator of a subset of Tcf/Lef family members, and can suppress secondary axis formation in *Xenopus* (Ghogomu et al., 2006). Important modulators of TCF/LEF activity are also found in the family of SOX (SRY-related HMG box) factors containing over 20 members (reviewed in Kormish et al., 2009; Bernard and Harley, 2010). In both mammalian cells and *Xenopus*, SOX17 and SOX4 directly bind to the HMG-box of TCF7l1, TCF7l2 and LEF1, an interaction that modulates the stability of the TCF/β-catenin complex (Sinner et al., 2007). More recently, SOX17 was shown to functionally cooperate with Wnt/β-catenin to specify an endodermal fate while repressing the meso-ectodermal fate. In this context, SOX17 and β-catenin co-occupy hundreds of key enhancers. In some cases, SOX17 and β-catenin synergistically activate transcription, apparently independently of TCF/LEF, whereas on other enhancers, SOX17 represses β-catenin/TCF-mediated transcription to spatially restrict gene expression domains. In this context, SOX17 acts as a tissue-specific modifier of the TCF/LEF responses (Mukherjee et al., 2021). Another modulator of the canonical Wnt signaling is SOX9, which was found to associate with β-catenin and further inhibit its activity (Topol et al., 2009). Further observations show that SOX9 proteins, together with Krüppel-like factor 4 (KLF4), suppress the Wnt-induced transcription through competing with TCF/LEF for the same β-catenin promoter sites, inhibiting the β-catenin-TCF/LEF (more specifically TCF7l2) binding and transcriptional activity (Sellak et al., 2012).

### Post-Translational Modifications Influence Both TCF/LEF and Gro/TLE Interactions

**FIGURE 5:**
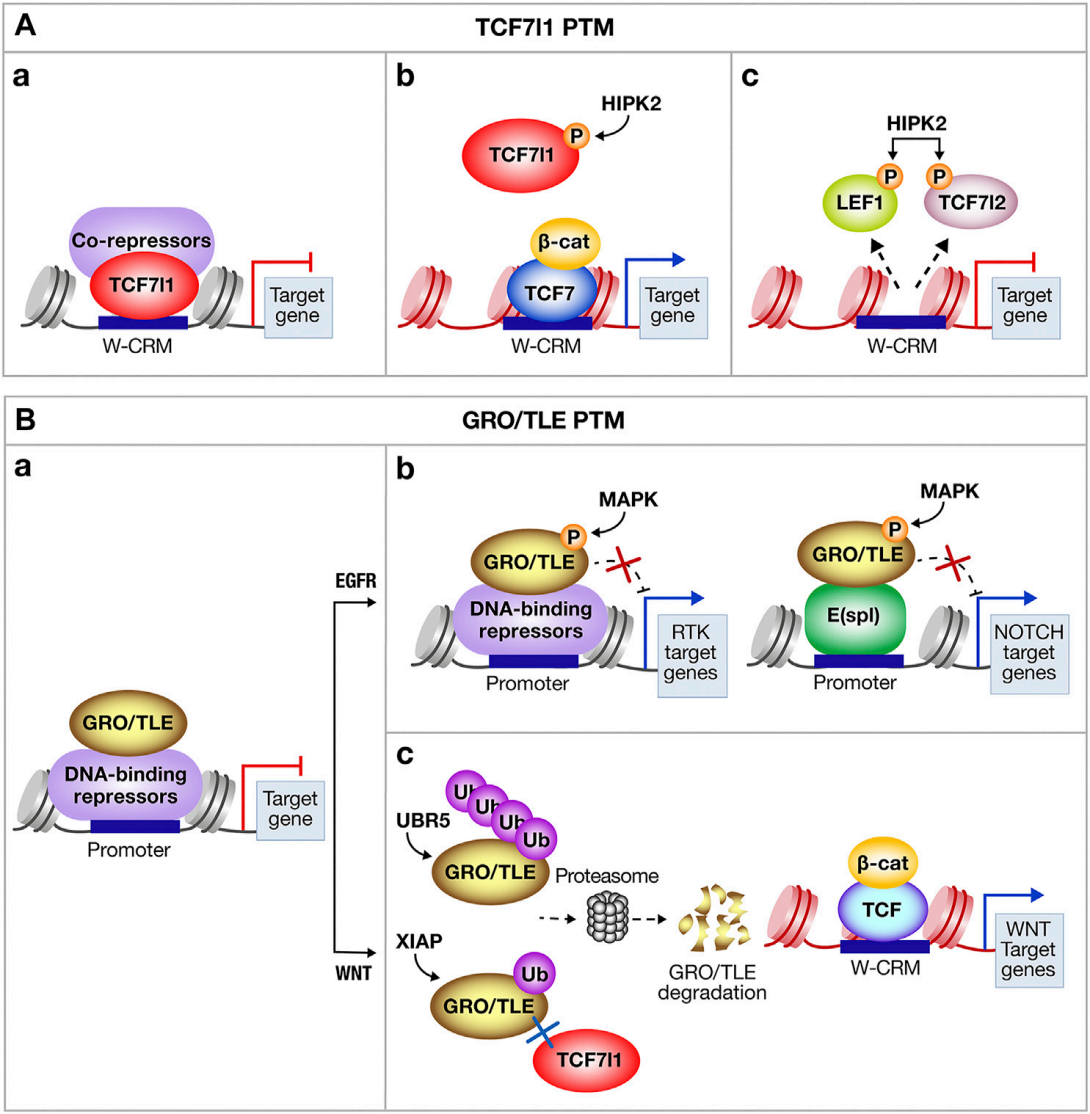
Some Post-Translational Modifications modulating the transcriptional activities of TCF7l1 and Gro/TLE. Post-translational modifications (PTM) including phosphorylation and/or ubiquitination of **(A)** T-cell factor-like-1 (TCF7l1) and **(B)** Groucho/Transducin-like enhancer of split (Gro/TLE) influence positively, or negatively, the transcriptional output of TCF7l1/Gro complex. **(A) (a)** TCF7l1 bound on W-CRM with co-repressors normally limits transcription. **(b)** The Homeodomain-Interacting Protein Kinase-2 (HIPK2) acts as a positive or negative regulator of Wnt target genes’ expression. Phosphorylation of TCF7l1 by HIPK2 decreases TCF7l1 affinity to target genes’ promoter and enables transcription through the β-catenin/T-Cell Factor-7 (β-cat/TCF7) complex. **(c)** Conversely, phosphorylation of the transcriptional activators Lymphoid Enhancer Factor-1 (LEF1) and TCF7l2 abolishes their binding to the promoter and blocks gene transcription. **(B) (a)** Gro/TLE together with DNA binding Co-repressor normally limits RNA Pol II mediated transcription. **(b)** The Receptor Tyrosine Kinase (RTK) phosphorylates Gro/TLE through the Mitogen-Activated Protein Kinase (MAPK) pathway, resulting in a decrease of Gro/TLE repressive activity. Gro/TLE mediates crosstalk between Notch and MAPK signaling pathways. Notch signaling activation leads to the expression of the Enhancer of split E(spl), which is a major transcriptional repressor of Notch target genes. E(spl) complexes with Gro/TLE to block target genes’ expression, including proneural genes. Phosphorylation of Gro/TLE by the MAPK pathway inhibits its function as a repressor. **(c)** When Wnt signaling is activated, the E3 ubiquitin ligase (UBR5) polyubiquitinates Gro/TLE in flies. Similarly, in vertebrate, the X-linked Inhibitor of Apoptosis (XIAP) is recruited to the transcriptional complex containing TCF7l1 and Gro/TLE, and monoubiquitinates Gro/TLE. Mono/polyubiquitination of Gro/TLE enables its degradation by the proteasome and blocks its re-association to TCF7l1, allowing the recruitment of the transcriptional co-activator β-cat to the activating TCF/LEF, and further expression of Wnt target genes. W-CRM, Wnt-Cis regulatory motif; EGFR, Epidermal Growth Factor Receptor.

Besides the spatial and temporal distribution of repressor partners, PTM, including ubiquitination and/or phosphorylation of TCF/LEF and Gro/TLE, influence positively or negatively, Gro/TLE-TCF/LEF interactions (reviewed in Cinnamon et al., 2008; Turki-Judeh and Courey, 2012; Ramakrishnan et al., 2018). In gastrulating *Xenopus* embryos and in mammalian cells, phosphorylation of TCF7l1 by the Homeodomain Interacting Protein Kinase 2 (HIPK2) inhibits its capacity to bind its target genes (Figure 5A). β-catenin was found to serve as a scaffold that promotes HIPK2 interaction with TCF7l1 and the subsequent dissociation of TCF7l1 from the target gene promoter, thereby opening the way for β-catenin interaction with the non-phosphorylated TCF7, and activation of Wnt target genes’ transcription (Figure 5Ab). Mutated TCF7l1 proteins resistant to Wnt-dependent phosphorylation function as constitutive inhibitors. HIPK2-dependent phosphorylation also causes the dissociation of LEF1 and TCF7l2 from their targets’ promoter (Figure 5Ac) and its effect is thereby highly context specific: HIPK2 up-regulates transcription by phosphorylating TCF7l1, a transcriptional repressor, but inhibits transcription by phosphorylating LEF1, a transcriptional activator (Hikasa et al., 2010; Hikasa and Sokol, 2011). Alternatively, in mouse embryonic stem cells (mESCs), β-catenin inactivates TCF7l1 by removing it from DNA, which is followed by TCF7l1 protein degradation. Interestingly, in this context, genetic cues indicate that TCF7l1 inactivation appears to be the only required effect of the TCF7l1/β-catenin interaction (Shy et al., 2013).

Gro/TLE co-repressors are targets of PTM, which modulate their affinity not only for Wnt effectors, but also Notch, and Epidermal Growth Factor Receptor (EGFR) signaling cascades (Figure 5B). One example comes from studies in *Drosophila* demonstrating that EGFR signaling, mediated *via* the Mitogen-Activated Protein Kinase (MAPK), phosphorylates Gro/TLE, and leads to the weakening of its repressor function, and attenuation of Gro/TLE-dependent transcriptional silencing by the E(spl) proteins, which are the effectors of the Notch cascade (Figure 5Bb). Reversibly, when RAS/MAPK signaling is impeded, Gro/TLE-mediated repression is enhanced both *in vitro* and *in vivo*. Thus, downregulation of Gro/TLE-dependent repression by MAPK modulates the transcriptional output of the Notch pathway, and possibly of other pathways (reviewed in Cinnamon et al., 2008). In both *Drosophila* and human cell lines, the E3 ubiquitin ligase UBR5 is required for Wnt cellular response. In this context, Wnt signaling induces the ubiquitination of Gro/TLE by UBR5, which happens downstream of β-catenin stabilization (Figure 5Bc). *In vivo* observations argue that ubiquitination inactivates Gro/TLE, thereby enabling Arm/β-catenin to activate transcription (Flack et al., 2017). Interestingly, inactivation of Gro3/TLE3 occurs *via* the activity of AAA ATPase Valosin-containing protein (VCP, also known as p97). VCP unfolds ubiquitinated proteins *via* its ATPase activity and disrupts ubiquitinated Gro3/TLE3 tetramerization, a process required for Gro/TLE to repress Wnt targets (Chodaparambil et al., 2014). Moreover mono-ubiquitination of Gro3/TLE3 by the E3 ubiquitin ligase XIAP (X-linked Inhibitor of Apoptosis) at its N-terminal Q-rich domain disrupts the ability of Gro3/TLE3 to bind TCF7l1, and consequently inhibits TCF7l1 repressor activity. XIAP is recruited to the Gro/TCF complex upon Wnt pathway activation, which enhances β-catenin/TCF complex assembly and the initiation of a Wnt-specific transcriptional activation program (Hanson et al., 2012). Because UBR5 and XIAP ubiquitinate Gro3/TLE3 in distinct ways (poly vs. mono) and at different locations on the Gro3/TLE3 protein, it is possible that the two E3 ligases modulate the Wnt transcriptional switch either in parallel, or simultaneously, depending on the cellular context. In addition to its ubiquitination activity, XIAP has been shown to play a role in inhibiting Caspases. In vertebrate, XIAP directly binds to and functionally blocks Caspase-3, Caspase-7 and Caspase-9 proteolytic activity (reviewed in Liston et al., 2003). However, there is no evidence for the XIAP-mediated degradation of vertebrate Caspases *in vivo*, which appears to depend on the type of ubiquitination and on the cell type.

### Evolutionary Conservation of BARHL Protein’s Structure and Functions

As previously described, BARHL1 and BARHL2 proteins have a strong degree of homology between one another. BARHL2 is highly conserved amongst distant species in the evolutionary scale, as observed through the high aa sequence conservation throughout its entire sequence (Figure 4). Besides, they are amongst the TFs essential for patterning the body axis of the developing embryo that are conserved in simpler organisms beyond the phylum of chordates. For example, genetic programs ancestral to the ones required for vertebrate development were found conserved in hemichordates. In *S. kowalevskii*, which is thought to be the closest species to the common ancestor at the base of the phylogenetic tree of chordates, *Barhl2* ortholog gene shares close similarities in its distribution and expression patterns compared to chordates (reviewed in Röttinger and Lowe, 2012; Sena et al., 2016). A conserved Shh Brain Enhancer (SBE1) has been discovered in mice with an equivalent function to that described in the *S. kowalevskii*. SBE1 directly regulates *Shh* expression in the *zli* through binding the second intron of the *Shh* gene. Diverse transcription factors, including Otx2 and Barhl2, directly regulate SBE1 within the *zli*. Functional analysis in both species demonstrated sufficient conservation between *Barhl2* and one of the *S. kowalevskii barH* HD for both binding, and activating CRM, thereby controlling *Shh* expression (Yao et al., 2016) (reviewed in Sena et al., 2016).

In *C. elegans*, the cephalic chemosensory neurons (CEM) undergo PCD during hermaphrodite embryogenesis but not in males (Sulston et al., 1983), a process relying on CEH30*,* a Bar-HD transcription factor (Schwartz and Horvitz, 2007). CEH30 protein interacts with UNC-37, which is the *C. elegans* homologue of Gro/TLE, through its N-terminal EH1 motif. It thereby prevents cell death (Peden et al., 2007) and inhibits transcription of *egl-1* gene, which encodes the executioner cell death protein CED-3, one of the major components of the PCD in worm (Nehme et al., 2010). Sequence comparison between human BARHL2 and CEH-30 proteins reveals 64% identical amino acids in the region including the HD and the motif immediately next to the HD on the C-terminal side called the BARC motif (Bar homeodomain C-terminal motif) (Figure 4). Interestingly, murine *Barhl1* or *Barhl2* genes compensate for the loss of function of CEH-30 in *C. elegans* (Schwartz and Horvitz, 2007), which is consistent with a conservation of *Barhl* genes’ function through evolution.

## The Core Role of TCF7l1 as a Transcriptional Inhibitor in Driving Embryonic and Neural Stem Cells Towards Differentiation

### In Mouse Embryonic Stem Cells, Inhibition of TCF7l1-Mediated Repression Promotes Self-Renewal and Pluripotency

**FIGURE 6:**
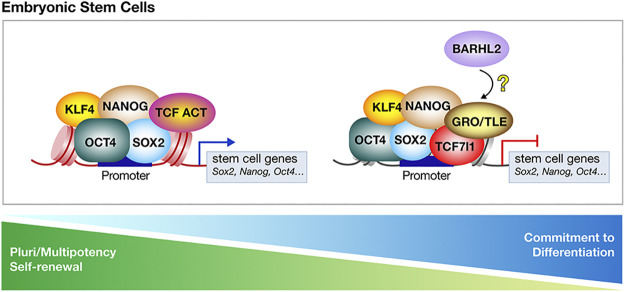
TCF7l1-mediated repression is at play in committed mouse embryonic stem cells (mESCs). In mESCs, the T-Cell Factor/Lymphoid Enhancer Factor (TCF/LEF) switch from a transcriptional activator to inhibitor, controls the balance between pluripotency and differentiation. Key pluripotency genes Octamer-binding transcription factor-4 (OCT4), SRY-box 2 (SOX2), NANOG and Krüppler-like factor-4 (KLF4) mark the pluripotent state of mESCs and are associated with the co-activators TCF7. In mESCs, TCF7l1 is the most expressed member of the Tcf/Lef family. TCF7l1 associates with regulatory regions that are bound by OCT4, SOX2, NANOG, and KLF4. Through interacting with Groucho/Transducin-like enhancer of split (Gro/TLE), TCF7l1 inhibits the expression of “stem cells” genes and allows mESCs to differentiate. Eliminating TCF7l1 repressive activity on mESCs pluripotency network allows the reacquisition of pluripotency and self-renewal. BARHL2 is expressed in mESCs during their commitment phase and could participate in driving mESCs towards irreversible commitment and differentiation by blocking TCF7l1 in a transcriptional inhibitory state.

mESC isolated from the inner cell mass of the blastocyst, the pre-implementation stage mammalian embryo, are characterized by their ability to self-renew and to differentiate into all types of somatic cells, a process referred to as pluripotency (reviewed in Chen et al., 2017). A specific core set of transcription factors including OCT4 (Octamer-binding transcription factor 4), NANOG, SOX2 and KLF4 form regulatory circuitry consisting of autoregulatory and feedforward loops thereby supporting pluripotency and self-renewal of these cells (Boyer et al., 2005). Extracellular signaling including LIF/JAK/STAT3 (Leukemia Inhibitory Factor/Janus Kinase/Signal Transducer and Activator of Transcription) (Williams et al., 1988; Ying et al., 2008), Wnt (Hao et al., 2006; ten Berge et al., 2011), BMP (Ying et al., 2003), and the MAPK/ERK (Yang et al., 2012) cascades, influence mESCs fate decision. Indeed, in mESC, Wnt signaling has been demonstrated to have important, somewhat difficult to interpret, activities (reviewed in Niwa, 2011; Merrill, 2012). From all these studies, a consensus emerges that inhibition of TCF7l1-mediated repression is at the core of mESC self-renewal and pluripotency (Figure 6) (Atlasi et al., 2013) (reviewed in Sokol, 2011; Wray and Hartmann, 2012). Reversibly, enhancement of TCF7l1 repressive activity blocks mESC self-renewal, and allows mESCs to differentiate, even in the presence of Wnt signaling (Wray et al., 2011).

In mESCs, TCF7l1 is the most expressed member among the TCF/LEF protein family (Pereira et al., 2006; Salomonis et al., 2010). Whole-genome approaches including RNA-seq and ChIP-seq show that TCF7l1 transcriptionally represses many genes important for maintaining pluripotency, and self-renewal, as well as those involved in lineage commitment, and stem cell differentiation. TCF7l1 associates with the regulatory regions of 1369 genes (Tam et al., 2008). Among those regions, 1173 bind TCF7l1 and OCT4 (Cole et al., 2008) with more than 940 binding TCF7l1, OCT4, SOX2, and NANOG (Boyer et al., 2005; Pereira et al., 2006; Marson et al., 2008). Depletion of TCF7l1 generates mESC’ refractory to differentiation (Cole et al., 2008). Moreover, both the Gro/TLE and CtBP interacting domains of TCF7l1 are required for *OCT4* repression (Tam et al., 2008). Finally, *KLF4* gene contains conserved TCF/LEF binding sites, and its expression is downregulated by TCF7l1 (Park et al., 2015). Interestingly, two TCF7l1 isoforms have been discovered, and are expressed equally in mESCs, where they regulate both an overlapping, as well as different sets of target genes. Removal of one of both TCF7l1 isoforms was found sufficient to stimulate self-renewal and delay the differentiation through repression of *NODAL* and *KLF4* (Salomonis et al., 2010). Further analysis revealed that binding of β-catenin to both TCF7l1 and TCF7 contributes to the maintenance of self-renewal and gene expression, at least partly through their recruitment to OCT4-binding sites on ESC chromatin (Yi et al., 2011).

The crucial role of TCF7l1 is reinforced by analysis of mESCs lacking all full-length TCF/LEF. In such cells, re-expression of TCF7l1 makes mESCs capable of differentiating into the three lineages, including neuronal cells (Moreira et al., 2017). In this context, TCF7l1 has been shown to directly interact with OCT4, and compete with SOX2 at some SOX-CRM, a process under the influence of MEK/MAPK (Zhang et al., 2013). Indeed, besides limiting TCF7l1-mediated repression of the pluripotency network, inhibition of the MAPK/ERK pathway participates in maintenance of pluripotency and self-renewal (reviewed in de Jaime-Soguero et al., 2018). In mESCs, inhibition of MEK suppresses *LEF1* expression, and depletion of LEF1 partially mimics the self-renewal-promoting effect of MEK inhibitors. In the absence of the exogenous factors, cytokines or inhibitors, depletion of both TCF7l1 and LEF1 enables maintenance of undifferentiated mESCs (Ye et al., 2017).

In agreement with all these data, Gro/TLE, more specifically Gro4, is not required for sustaining pluripotency, and suppressing differentiation genes in mESC. Rather, Gro/TLE activity appears necessary for early differentiation where it acts to suppress the pluripotency network, allowing for the initiation of lineage specific gene expression programs. In mESCs, most of the genes occupied by TCF7l1 were found co-occupied by Gro/TLE (Laing et al., 2015). Through interacting with Gro/TLE, TCF7l1 represses *NANOG* (Pereira et al., 2006), and repression of *OCT4* was found to rely on TCF7l1/Gro2 interactions (Tam et al., 2008). Interestingly, the dominant-negative GRG5 is highly expressed in mESC, and its expression drops once mESCs exit the pluripotent state, to increase again during neuroectodermal cell specification. Whereas overexpression of *GRG5* promotes self-renewal, its siRNA-mediated KD deregulates the mESC pluripotent state. Transcriptomic analysis reveals that, in this context, GRG5 represses mesendodermal-related genes, and promotes neuronal specification *via* inhibition of Wnt and BMP signaling. Moreover, GRG5 maintains the self-renewal of NSCs by sustaining the activity of Notch/HES and STAT3 signaling pathways (Chanoumidou et al., 2018).

In contrast to what is reported in mESC, in human ESC (hESCs), Wnt/β-catenin signaling appears to be actively repressed in an OCT4-dependent manner during self-renewal. In these cells, activation of Wnt signaling appears to induce loss of self-renewal, and differentiation into mesodermal lineages (Davidson et al., 2012). Although such discrepancy is a little puzzling, it has been shown that generation of neural lineages from hESCs requires inhibition of Wnt signaling (Tabar and Studer, 2014) and that activation of Wnt signaling in hESCs-derived neural precursor cells promotes transcription of midbrain-like genes through TCF7l2 directly binding the *Engrailed-1 (EN1)* promoter (Kim et al., 2018).

### The Case of Neural Stem Cells During Development

Besides its role in development of the CNS, Wnt/β-catenin signaling is crucial for NSCs maintenance. NSCs emerge from territories that have kept their neuroepithelium properties and respond to Wnt signals from embryogenesis through adulthood (Selvadurai and Mason, 2011; Garbe and Ring, 2012; Borday et al., 2018). In the subventricular zone of the developing mouse brain, Wnt signaling is a hallmark of self-renewing, specifically of NSCs’ (Kalani et al., 2008). Investigation of the developmental fate of Wnt/β-catenin–responsive cells in embryonic and postnatal mouse brain using a reporter for *Axin2*, demonstrates the continued importance of persistent Wnt/β-catenin signaling for NSCs and progenitor cells emergence (Bowman et al., 2013). In mouse adult hippocampus, where new neurons are continuously generated from NSCs, expression of the pro-neural TF Neurogenic Differentiation 1 (*NEUROD1*) is a landmark of cells dropping out of self-renewal and entering neuronal commitment. Overlapping binding sites for the TCF/LEF factors and SOX2, a marker of most uncommitted cells of the CNS, are present in the promoter region of *Neurod1*. In this context, Wnt signaling together with removal of SOX2 triggers the expression of *NEUROD1*, demonstrating that the SOX2-TCF/LEF regulatory elements are critical for *NEUROD1* expression, and consequently for the switch from the SOX2-mediated repression to the TCF/LEF-mediated activation, towards a neuronal fate ((Kuwabara et al., 2009). In neural precursor cells of the mouse’ neocortex, expression of TCF7l1 was found to repress Wnt activity (Ohtsuka et al., 2011; Kuwahara et al., 2014), and active Wnt signaling in the rodents’ neocortex apical progenitors sustain their fate plasticity (Oberst et al., 2019). In cultured rat adult hippocampal NSCs, fate decision is influenced by the temporal variations of β-catenin. Optogenetic approaches reveal that continuous activation of β-catenin in cultured NSCs specifies neuronal differentiation, whereas short β-catenin signals activate proliferation but remain insufficient to induce neuronal differentiation. Loss of β-catenin signals promotes apoptosis in differentiating cells, which could be due to inappropriate cell-cycle re-entry (Rosenbloom et al., 2020).

### BARHL2 Promotes mESCs Differentiation

Does BARHL2 play a part in mESC biology *via* its ability to enhance TCF7l1 repressive activity*?* Although such a question has not been directly asked, it is known that *BARHL2* is expressed in mESCs during their commitment phase (Lee et al., 2006). Global expression profile analysis of mESC lines in which BARHL2 overexpression was induced in a doxycycline-controllable manner, reveals that BARHL2 induces a significant fold-change in more than three thousand genes with more than two thousand genes being upregulated, and more than one thousand genes downregulated. In this context, BARHL2 was one of the most influential TF analyzed. Two days following BARHL2 induction, mESCs start to express mesodermal lineage markers (Yamamizu et al., 2016). In a study using another BARHL2 overexpression design in mESCs, a significant increase in the population of neural cells was observed 14 days post-induction (Teratani-Ota et al., 2016). In this context, Notch signaling pathway played a significant influence in driving neural differentiation, and the majority of neuron-like cells generated by induction of BARHL2 expressed markers of GABAergic neurons.

Work is still needed to understand the context-specific regulation of TCF/LEF activities in the biology of ESC, specifically in hESCs. However, studies from the last 15 years strongly support inhibition of TCF7l1-repression as the necessary downstream effect of Wnt signaling in the promotion of mESCs’ self-renewal and pluripotency. Reversibly, the formation and stabilization of the TCF7l1/Gro complex, and its inhibitory influence on specific chromatin loci, is one of the crucial switches driving ESCs towards cellular commitment (reviewed in Sokol, 2011). Genetic and functional studies demonstrated that the Gene Regulatory Networks (GRN) underlying acquisition/loss of the pluripotent state are similar in the rodent, fish, and amphibian’s blastomeres, and in mESCs, with slight differences observed in hESCs. In early blastomeres, BARHL2-mediated lock of TCF7l1 in an inhibitory state pushes early SO cells towards irreversible commitment and differentiation, arguing for a similar function in mESCs.

### Wnt Signaling Deregulation and Stem Cells: The Emergence of Cancer

In the past years, an increasing number of studies have demonstrated that mutations, loss, or aberrant regulation of Wnt signaling are at the origin of a wide variety of diseases (reviewed in Noelanders and Vleminckx, 2017; Ng et al., 2019). In one of its severest forms, Wnt constitutive activation is associated with diverse cancer types including melanoma, leukemia, breast cancer, gastro-intestinal cancers, and others (reviewed in Zhan et al., 2017). Cancer Stem Cells (CSCs), also known as Tumor Initiating Cells (TICs), are characterized by their “stemness” characteristics that contribute to tumor progression and drug resistance and play deterministic roles in cancer recurrence. Cancer cells exhibit many of the same features as stem cells including self-renewal and their low level of differentiation. Whereas the exact connection between cancer and stem cells is not completely understood, it is well established that both cells use similar signaling pathway machineries, specifically those of the Wnt/β-catenin, Shh, MAPK/ERK and Notch pathways (reviewed in Friedmann‐Morvinski and Verma, 2014). In this section, we focus on the impact of Wnt signaling on CSCs, and on medulloblastoma (MB), a pediatric tumor of cerebellar origins in which contributions of both TCF/LEF and the BARHLs are relevant.

### Wnt and Cancer Stem Cells

As observed through activity of a TCF/LEF reporter gene, Wnt/β-catenin signaling is highly active in various types of CSCs including colon, lung, breast, and gastric cells (Vermeulen et al., 2010; Horst et al., 2012). A pharmacological antagonist of β-catenin/TCF7l2 interaction blocks CSCs’ self-renewal and suppresses tumorigenesis. Treatment of human colon cancer cells, and mouse salivary gland cells with such compound did not only reduce the β-catenin/TCF7l2-mediated proliferation rate and self-renewal, but also induced the differentiation of tumor cells (Fang et al., 2016), making it a potential therapeutic target of the β-catenin-TCF/LEF-dependent tumors, among other tested drugs (reviewed in Jung and Park, 2020; Walcher et al., 2020; Zhang and Wang, 2020). Non-coding RNAs have also been identified as modulators of Wnt-TCF/LEF activity in CSCs. For example, miR-142, which is absent in normal mammary cells but highly expressed in mammary CSCs, increases Wnt activity by inducing degradation of APC, a negative regulator of canonical Wnt signaling (Isobe et al., 2014). Additionally, the long non-coding RNA IncTCF7 has been characterized in hepatocellular carcinoma cells. IncTCF7 maintains CSCs’ properties *via* TCF7-dependent activation of Wnt signaling (Wang et al., 2015). Notably, the ability of both normal and CSCs to maintain long telomeres – an important feature to prevent their cellular aging – appears to be under direct transcriptional control of β-catenin-TCF/LEF (Park et al., 2009; Noureen et al., 2021). Whereas the promoter of the telomerase enzymatic subunit, *TERT*, neither bind TCF7l2 nor TCF7l1, it is enriched with TCF7-binding sites located close to the transcription start site that binds β-catenin specifically (Hoffmeyer et al., 2012).

### Medulloblastoma

Medulloblastoma (MB), the most common childhood malignant brain tumor, emerges from the cerebellum and accounts for 30% of pediatric brain tumors. Integrated genomic studies allowed the identification of four types of human MB, corresponding to specific genetic alterations (reviewed in Hatten and Roussel, 2011; Wang et al., 2018). One group is associated with alterations in the Wnt/β-catenin signaling pathway (15% of the cases) and originates from brain stem cells. A second group (25% of the cases) is characterized by the constitutive activation of the Shh pathway and derives from Granule Neuron (GN) progenitors. Group 3 (30% of the cases) is specifically found in infants and is thought to originate from overexpression of the MYC oncogene in cerebellar NSCs. Whereas group 4 is the most common MB subgroup (30% of the cases), its underlying biology is not well understood.

In the rodent brain, the cerebellar upper Rhombic Lip (uRhL) produces the GNs that constitute the largest neuronal population in the brain. The GN population exhibits a unique developmental trait: committed GN progenitors (GNPs) are characterized by a very long period of “quiescence” occurring before birth, followed by a long proliferative phase – i.e., 2 weeks in mouse, 2 years in human - occurring after birth, before their final differentiation step (reviewed in Leto et al., 2016). Due to this developmental specificity, this cell population is at risk when it comes to the appearance of developmental defects, including oncogenic events (reviewed in Hatten and Roussel, 2011). At birth, the RhL stem/progenitor cells become responsive to secreted SHH that stimulates their proliferation and self-renewal. The uRhL exhibits stem cell niche properties and exhibit positive TCF/LEF transcriptional activity (Selvadurai and Mason, 2011; Bowman et al., 2013; Yeung et al., 2014; Borday et al., 2018)**.** Atonal Homologue 1 (ATOH1/MATH1) is the master gene of GNPs’ development (reviewed in Leto et al., 2016). ATOH1 directly induces the expression of *Barhl1* and *Barhl2* (Kawauchi and Saito, 2008). A thorough single-cell RNA-seq performed on mouse cerebellar cells reveals that *Tcf7l1* is expressed strongly early in the GNPs’ differentiation pathway (Wizeman et al., 2019), and that *Barhl2* expression is uniquely associated with early fate commitment in the GNPs (Carter et al., 2018). Taken together, these observations argue that BARHL2 could participate in driving GN stem/progenitor cells towards irreversible commitment.

Rodent cerebellar uRhL cells are known to be at the origin of group 2 MB that are associated with deregulation of the Shh pathway (reviewed in Hovestadt et al., 2020; Garcia-Lopez et al., 2021). Some tumor-propagating cells from this subgroup are not only resistant to Shh inhibitors but are also TCF/LEF-dependent for their self-renewal (Rodriguez-Blanco et al., 2017). *in silico* analysis associates *BARHL2* expression with the emergence of group 2 MB, and *BARHL1* expression with emergence of group 3 and group 4 MB (Pöschl et al., 2011; Lin et al., 2016). Taken together, these observations are a good starting point for future research that should evaluate whether BARHLs act as roadblocks for de-differentiation that are corrupted in MB.

## Conclusion and Perspectives

In this review, we provide an overview of the TCF/LEF activities as transcriptional repressors focusing on the highly evolutionarily conserved roles of Wnt signaling in axis establishment, neural proliferation, and stem cell biology. We also described the importance of the pro-neural TF BARHL2 as an enhancer of TCF7L1 repressor activities in both SO formation, and forebrain progenitor proliferation.

Currently, numerous conundrums regarding the developmental regulation(s) of TCF/LEF activities, including their interactions with the Barhls, are unresolved. An important point is to clarify the interactions between Barhl2, and generally the Barhls, with the different TCF/LEF members: which Barhl interacts with each of the TCF/LEF family? Are these interactions specific to the different TCF/LEF isoforms and their splice variants? What characterizes the TCF/LEF-BARHL binding interface? Is the interface evolutionarily conserved? Numerous signaling pathways interplay to orchestrate the multipotency/commitment/proliferation states of neural stem/progenitor during embryogenesis. Besides Wnt signaling, the activation and/or inhibition of TCF/LEF activity is under the influence of Notch, Shh, and MAPK/ERK pathways. The context, the repression partners, and the PTM involved in controlling these subtle levels of regulation in NSCs are still poorly understood. Exploration of *Barhl2* developmental expression indicates that it is either a direct, or an indirect, target of the canonical Wnt signaling pathway, thereby contributing to the establishment of a negative feedback loop, that limits the TCF/LEF transcriptional activity in neural progenitors. Analysis of TCF/LEF activity in pluripotent versus committed ESCs indicate that TCF/LEF mostly act by changing the chromatin state in such a way that the expression of the pluripotency-related genes is switched off (reviewed in Sokol, 2011). As BARHL2 blocks TCF7L1 in a transcriptional inhibitory state, it is tempting to speculate that BARHL2 participates to driving stem/progenitor cells towards irreversible commitment, thereby establishing a roadblock on the cell trajectory towards differentiation.

Another important unresolved question relates to how BARHL2, TCF/LEF and Gro/TLE act long distance to transcriptionally inhibit key “commitment” genes. Understanding the specificity of BARHL2 DNA-binding alone, or together with TCF/LEF, should be a first step in identifying the set of genes whose expression is silenced *via* BARHL2/TCF7L1 activity. Moreover, understanding how the BARHL2/TCF7L1 modulates the open/close state of the chromatin, together with probable roles of PTM on the complex stability and its transcriptional activity, shall prove quite beneficial beyond understanding early embryogenesis. Finally, in both amphibian, and rodent, Barhl2 participates in the formation of the caudal forebrain organizer, partly through its direct activation of *Shh* transcription together with *Otx2* (Yao et al., 2016) (reviewed in Sena et al., 2016). Thereby, BARHL2’s function is not strictly restricted to its activity as a Wnt transcriptional repressor, but probably depends on the cellular context, adding another level of complexity that should be taken into consideration.
